# Implicit membrane for helical peptide selectivity toward bacterial membranes

**DOI:** 10.1016/j.bpj.2026.03.059

**Published:** 2026-04-06

**Authors:** Sofía Blasco, Erin Spearing, Martina Drabinová, Vendula Rašková, Robert Vácha

**Affiliations:** 1 CEITEC – Central European Institute of Technology, Kamenice 5, 625 00 Brno, Czech Republic; 2 National Centre for Biomolecular Research, Faculty of Science, Masaryk University, Kamenice 5, 625 00 Brno, Czech Republic; 3 Department of Condensed Matter Physics, Faculty of Science, Masaryk University, Kotlářská 2, 611 37 Brno, Czech Republic

## Abstract

Membrane lipid composition varies significantly across organisms, cell types, and organelles. Mammalian membranes predominantly contain lipids like phosphatidylcholine or sphingomyelin, whereas bacterial membranes are rich in phosphatidylglycerol, phosphatidylethanolamine, and cardiolipin. This diversity in lipid composition presents an opportunity to design peptides that target specific cell types. Particularly, peptides designed to preferentially bind bacterial membranes can have applications to treat bacterial infections while avoiding toxicity. Here, we present a method to identify a broad range of peptide sequences with preferential binding to bacterial membrane models. Using molecular dynamics simulations, we calculated the free energy of insertion for natural amino acid side chains into simplified bacterial and mammalian membranes and implemented a genetic algorithm to identify alpha helical peptide sequences that preferentially adsorb to bacterial membranes. The main limitation of the model is the assumption of helical secondary structure.

## Graphical abstract

**Figure:**
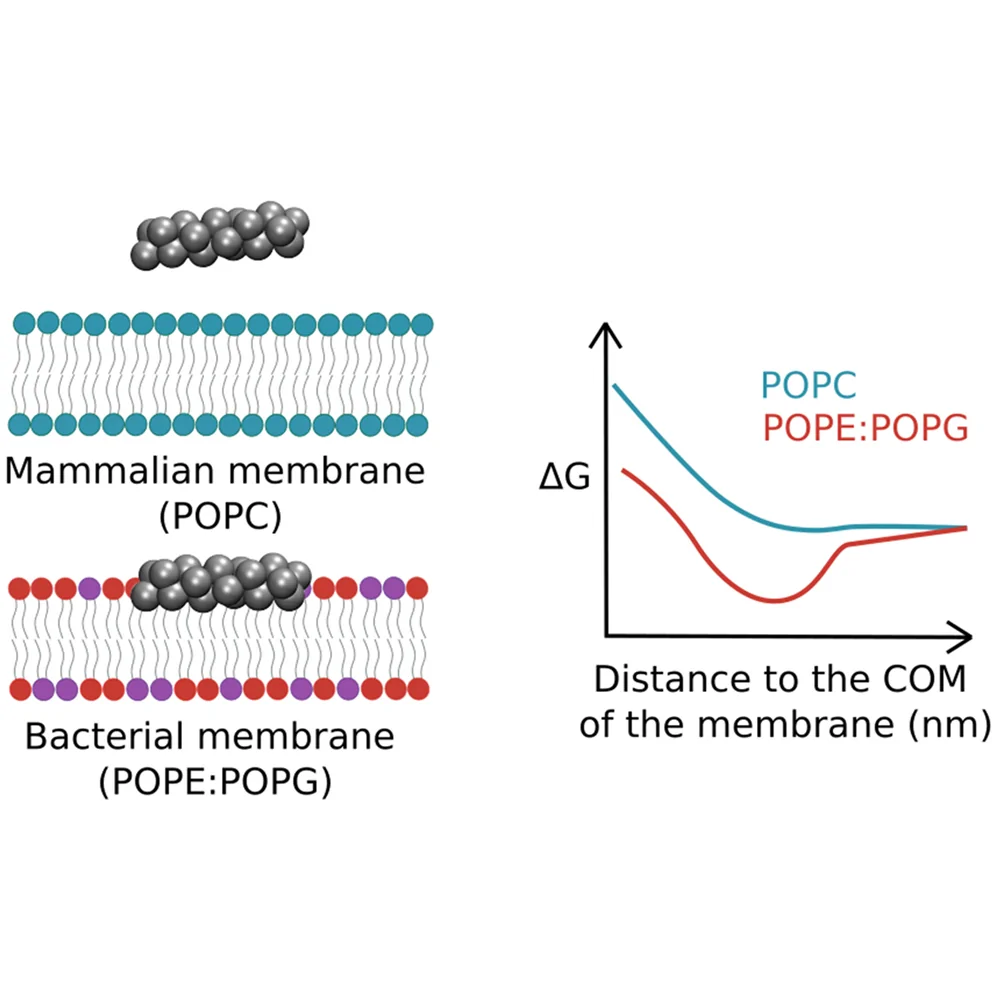

## Significance

This study presents a computational framework for designing alpha helical peptides that selectively target bacterial over human membranes, addressing the need for safer antimicrobial agents. We developed an implicit membrane model based on amino acid insertion energies, capturing effects beyond simple electrostatics of charged and uncharged lipids. Coupled with a genetic algorithm, the model identified peptide sequences with strong bacterial selectivity. Molecular dynamics simulations and affinity measurements validated the predictions. This approach enables rational peptide design, offering a pathway to antimicrobial therapeutics with reduced host toxicity.

## Introduction

Lipids are the principal constituents of cell membranes, forming a lipid bilayer that separates the inside and the outside of the cell. The lipid composition of cell membranes varies between organisms, cell types, or organelles (1). For instance, mammalian cell membranes are mainly composed of phosphatidylcholine (PC), sphingomyelin, phosphatidylserine, and phosphatidylethanolamine (PE) lipids (2,3,4), while bacterial cell membranes are rich in phosphatidylglycerol (PG), PE, and cardiolipin (5,6).

Diversity in lipid compositions of cell membranes provides an opportunity for the design of peptides that can selectively bind to specific cell types. In particular, peptides with preferential binding to bacterial membranes compared with mammalian membranes can be used for targeted drug delivery to bacteria, while avoiding toxicity in mammalian cells. Drug delivery to bacteria can be achieved, for example, by linking an antimicrobial drug to a bacterial selective peptide (7) or decorating the surface of liposome delivery systems with bacteria-selective peptides (8,9). Similarly, peptide sequences with membrane selectivity can be used to modify membrane-active peptides, such as antimicrobial peptides or cell-penetrating peptides, so that they only interact with the desired membrane type.

Peptide selectivity has been widely studied in the context of membrane-active peptides (10,11,12). However, peptide selectivity is often described in terms of differential membranolytic activity. This membranolytic activity of the peptide not only depends on the binding affinity of the peptide to the membrane, but also on peptide-peptide interactions and their ability to induce structural changes in membranes, e.g., pores. Therefore, to fully understand peptide selectivity, it is necessary to separate peptide selective affinity to the membrane and the mechanism of action that occurs once the peptide is adsorbed.

In this work, we develop a method to find alpha helical peptide sequences with binding selectivity toward lipid headgroups that are abundant in bacterial membranes, specifically those of the inner membrane of Gram-negative bacteria (PE and PG). We used molecular dynamics (MD) simulations to calculate the free energy of insertion of each amino acid side chain into simplified models of bacterial and mammalian cell membranes and used a genetic algorithm to calculate sequences that preferentially adsorb to the bacterial membrane model.

## Materials and methods

### Molecular dynamics simulations

MD simulations were performed using GROMACS version 2020.3,(13) CHARMM36m protein force field (mar2019 version) (14) with tip3p water model, and CHARMM36 lipid force field.(15) The temperature was maintained at 310 K using velocity rescaling thermostat with a stochastic term correction (V-rescale) (16), and with a coupling constant of 1.0 ps. Two separate temperature baths were coupled to protein-lipid and water-ions groups to achieve the adequate temperature distribution. The pressure was kept at 1 bar using the Parrinello-Rahman barostat (17) and semiisotropic coupling. The coupling constant was set to 12 ps. The cut-off for coulomb and van der Waals interactions was set at 1.2 nm. Computing of electrostatic interactions was performed with the smooth particle mesh Ewald method. The time step was set to 2.0 fs.

Each system was composed of a lipid membrane of 400 lipids, around 35,000 water molecules, the necessary NaCl ions to neutralize the system and reach the physiological concentration of 0.15M, and the corresponding amino acid side chain analog. Energy minimization of each system was performed using the steepest descent algorithm. After minimization, it was equilibrated in several steps for a total equilibration time of almost 2 ns.

We used CHARMM-GUI (18) to build the two membrane models we used. To mimic bacterial and mammalian membranes we used model membranes composed of their main lipid components (POPE:POPG (3:1)) for bacterial membranes and POPC for mammalian membranes. These membrane compositions have been broadly used as models for such cell membranes and are commonly used to simplify the modeling of these membranes while maintaining their main characteristics (4,19,20).

Amino acid analogs were constructed by truncating the residue at the beta carbon and substituting the alpha carbon by a proton. The partial charge on the beta carbon was adjusted to maintain the total charge of each residue. Proline and glycine analogs were not created due to their structure. We also avoided histidine because of the complexity of its three protonation states. Simulations of charged amino acids were performed in their neutral and charged states.

Additionally, we prepared salt bridge analogs by simulating together positively and negatively charged side chains. To keep these analogs close enough during the simulation, a harmonic restraint was added with strength constant set to 3000 kJ/mol nm^2^ and *r*_0_ of 0.1 nm between the end hydrogen and oxygen of each side chain. We did the same for aromatic interactions and simulated aromatic side chains together.

We performed umbrella sampling (US) using the distance to the center of the membrane as the collective variable and calculated the potential of mean force (PMF) of each analog along the membrane axis. For each US, we pulled the analog inside the membrane and generated 31 windows separated 0.1 nm from each other. The pulling of the analogs was done from a starting point at 3 nm from the center of the membrane to the center of the membrane, which was defined using the local center of mass (COM) inside a cylinder of radius 1 nm around the analog. The cylinder was used to define the local COM of the membrane to provide a more accurate distance from the membrane center. Pulling rate was 5e−5 nm/ps, and the force constant was 2000 kJ/mol nm^2^. Each umbrella window was simulated for up to 600 ns. Then, WHAM (21) was used to calculate the PMF of each analog. To estimate the error bars of the PMFs we used the Bayesian bootstrap method implemented in WHAM and used 200 bootstraps.

To make sure that the resulting PMF did not depend on the initial configuration of the umbrella windows, and that we had reached a converged result, we also pulled some of the analogs in the opposite direction. The pull was from the center of the membrane at 0 nm until the water phase at 3 nm. We compared the resulting PMFs for alanine, lysine, and tryptophan in both pulling directions. The profiles obtained were the same within 3 kJ/mol variation. The only exception occurred with charged lysine at the center of the membrane (between 0 and 0.3 nm), where big differences were observed that were caused by the orientation of the side chain toward one leaflet or the other.

### Implicit model

**Figure 1:**
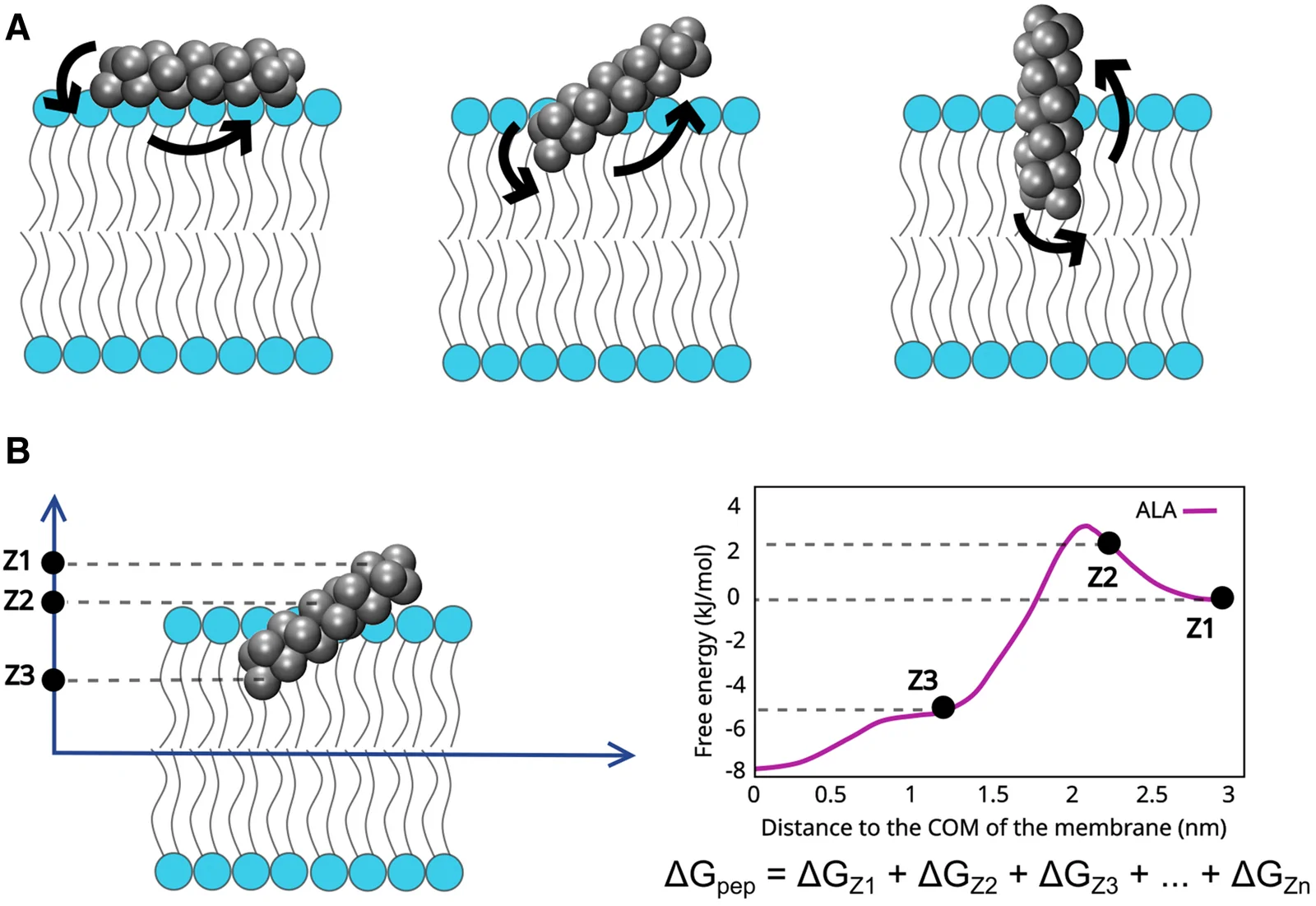
Illustration of different orientations of the peptide in the implicit membrane for its energy calculation. (*A*) In the three orientations shown in the figure, the center of mass (COM) of the peptide with respect to the membrane COM is the same. Through Boltzmann averaging of all orientations, we obtain the peptide free energy at specific peptide-membrane distance. (*B*) Example of calculation of the energy of the peptide in one orientation. We obtain the energy contribution from each amino acid from its z-position (distance from membrane COM) in that orientation. Three alanine residues at z-positions Z1, Z2, and Z3 are depicted together with corresponding positions at the free energy profile providing the free energy contributions. The total free energy of the peptide is the sum of all amino acid contributions from their specific positions in the calculated orientation.

We developed an implicit membrane model for mimics of human and bacterial membrane to assess the different affinities of the peptide sequences to these membranes. The implicit model calculates the energy of a peptide sequence from the free energy contributions of its constituent side chains. Affinity profiles are calculated as the sum of the free energy profiles for individual residues. For selected residue pairs (salt bridges and aromatic stacking interactions), we also added a correction for correlation of neighboring residues. In each distance of the peptide from the membrane COM, we calculate the energy for all peptide orientations; see Fig. 1. The energy contributions from orientations are then averaged out using Boltzmann weighted average leading to the final free energy profile of the peptide at each membrane.

### Genetic algorithm

We used a genetic algorithm to predict peptide sequences that would be selective to bacterial membranes. We set the length of the peptides to 20 amino acids and assume the peptides to have alpha helical conformation. The steps of the genetic algorithm include initialization, energy calculation, fitness evaluation, crossover, and mutation.

In the initialization step, an initial batch of sequences is generated to initiate the algorithm. We generated 100 random sequences for initialization.

In the energy calculation step, we use the implicit model to calculate the free energy profiles of the peptide for POPC and for POPE:POPG membrane.

In the fitness evaluation step, the energy profiles of each sequence are used to evaluate their fitness, according to a fitness function. We used as fitness function the difference between the ΔG minimum of each sequence in POPC and POPE:POPG membrane:

(1) $$\Delta \Delta G=\Delta G_{\mathrm{minPC}}-\Delta G_{\mathrm{minPE:PG}},$$

where Δ*G*_*minPC*_ is the energy minimum of the free energy profile in POPC membrane, and Δ*G*_*minPE:PG*_ is the energy minimum in POPE:POPG membrane. The higher the value of ΔΔ*G*, the fitter is the sequence. The four sequences evaluated as fittest are selected from the batch of sequences to continue to the next step.

In the crossover step, the first and third fittest sequences are crossed over between two random points, and the same is done with second and fourth fittest sequences. The mixed sequences are added to the population for the mutation step. At this point the sequence batch is formed by the four fittest sequences and two crossed over sequences.

In the mutation step, a random point in each sequence is selected to be mutated. Then, the residue at that point is mutated to all possible amino acid substitutions. An additional energy calculation and evaluation step is performed to select the fittest amino acid mutation on each sequence, and the fittest mutation on each sequence is added to the batch of sequences to be tested in the next step.

After mutation, the batch of sequences enters the energy calculation step and continues into a new cycle of the algorithm.

**Figure 2:**
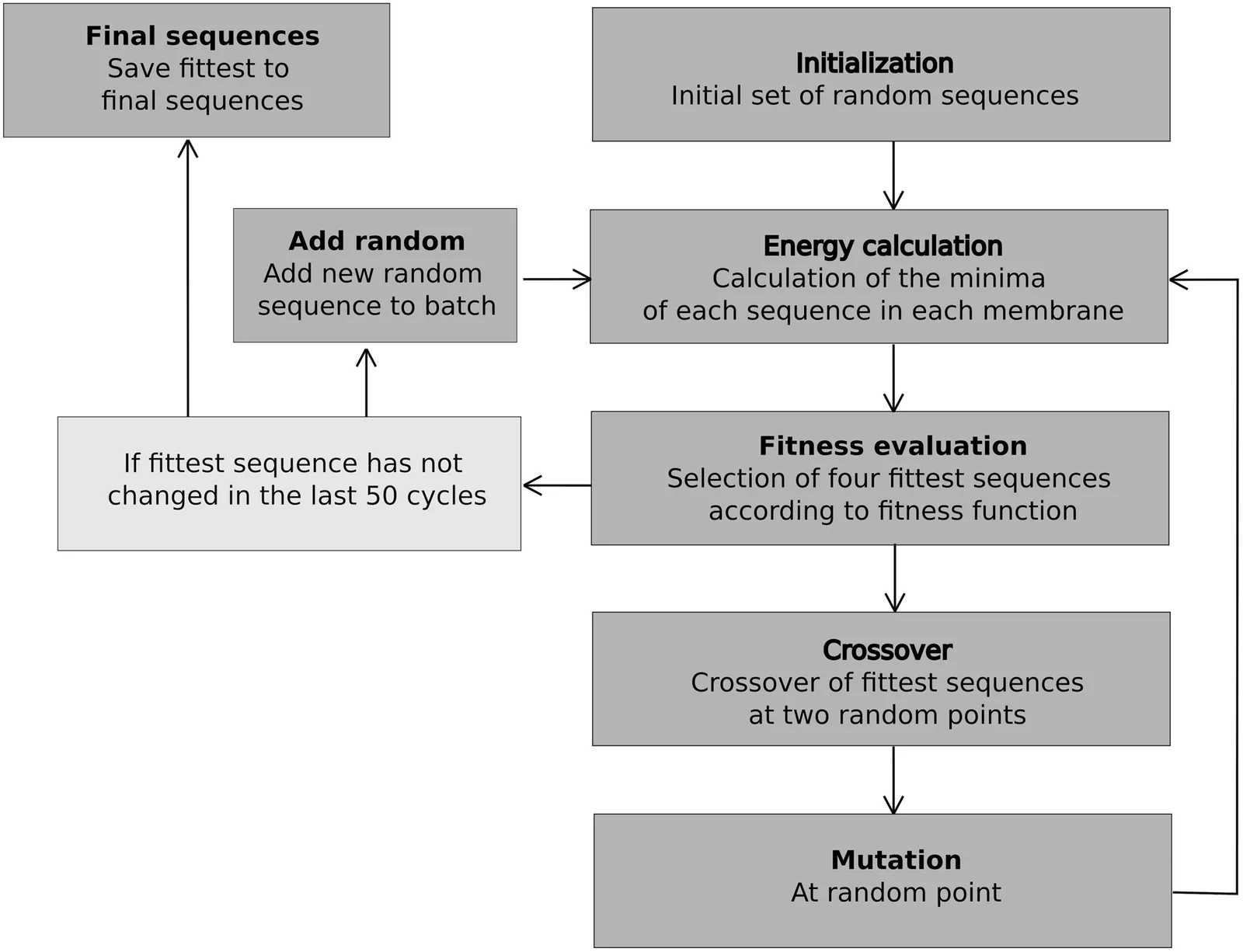
Schematic representation of the loop followed by the genetic algorithm

When the fittest sequence does not change after 50 cycles of the algorithm, that sequence is removed from the batch and saved as a final sequence. In its place, a new randomly generated sequence is added, and the algorithm continues until again; the fittest sequence does not evolve and is saved. The algorithm stops when we have saved as many final sequences as we want. A scheme of the complete workflow of the genetic algorithm is shown in Fig. 2.

After we obtained a set of sequences predicted to be selective toward bacterial membrane (sequences with the highest ΔΔ*G*), we also calculated the helicity of each sequence. The sequences with higher alpha helical content match the predicted values more closely because we assume the peptides to be in helical conformation in the energy calculations. We used the Jpred4 online server (22) to calculate the helicity of the peptides and selected the ones with the highest content and highest ΔΔG predicted.

Finally, we confirmed our results by calculating the PMFs of desorption of the selected peptides using US simulations. We initially placed the selected peptide at 1.5 nm from the membrane center and pulled it out to a distance of 4 nm from the membrane center. We restrained the alpha helical conformation during the simulation. We initially placed it parallel to the membrane plane, and we did not restrain the orientation of the peptide during the simulation, so it could reorient as it prefers. We generated 26 windows separated by 0.1 nm and sampled each window for up to 1200 ns.

We additionally calculated the PMFs of few pentapeptides for comparison. We built peptides five amino acids long composed of alanine and serine and performed the US simulations using the same protocol as for the side chain analogs. We restrained the alpha helical conformation of the peptide, so we can compare to our model. Nevertheless, the comparison is not ideal due to the backbone atoms available for hydrogen bonds. Longer peptides would reduce this effect but would cause larger membrane deformations, which the model cannot capture. Pentapeptide was thus selected as a compromise. For simplicity, we kept the peptide parallel to the membrane surface, thus avoiding the peptide tilting and rotation degrees of freedom. To keep the peptide parallel to the membrane, we restrained the movement of the alpha carbons in the x and y directions using position restraints. We used the PMFs of the pentapeptides to calculate the backbone contributions by subtracting the sum of the side chain PMFs to the pentapeptide PMF, as shown in Fig. S2.

We also computed backbone contributions using tryptophan and lysine pentapeptides and incorporated these contributions into the total energy calculations for selected 20 amino acid peptides. Backbone contributions were first derived at the single-residue level (alanine, serine, tryptophan, or lysine) by taking one-fifth of the pentapeptide free energy and subtracting the corresponding side-chain contribution. We assigned alanine-derived backbone contribution to hydrophobic residues, serine-derived contribution to polar residues, tryptophan-derived contribution to aromatic residues, and lysine-derived contribution to charged residues.

### QCM-D

The interaction of peptides with supported lipid bilayers composed of POPC and POPE:POPG (3:1 mol:mol) was assessed using quartz crystal microbalance with dissipation monitoring (QCM-D). This technique allows real-time detection of mass changes on the surface of a piezoelectric sensor oscillating at a frequency (f). The resonance frequency is dependent on the mass of the layer adsorbed onto the sensor surface, enabling measurement of the molecule-surface interaction. In this context, the binding of peptide to a lipid bilayer formed at the sensor surface would result in a negative frequency change. All experiments were conducted on a QSense Analyzer (Biolin Scientific, Sweden) equipped with SiO2-coated sensors (QSX 303, Biolin Scientific, Sweden). All reagents were added at a flow rate of 50 *μ*L/min.

POPC bilayers were formed by the spontaneous rupture and fusion method. Lipid films were prepared by adding 20 *μ*L of POPC (25 mg/mL in chloroform; Avanti, USA) to a round-bottom test tube, followed by solvent evaporation under a gentle stream of air and subsequent vacuum drying for 4 h. The films were stored at −20°C until use. On the day of the measurement, the lipid film was resuspended in 1 mL of PBS (pH 8.5) and sonicated on ice using a probe sonicator (60% amplitude, 10-s pulses) for 10 min until the lipid suspension became clear, indicating the formation of small unilamellar vesicles (SUVs). For the measurement, a baseline was established by flowing PBS (pH 8.5) through the analyzer for 10 min. SUVs were then added, followed by another 10 min of PBS (pH 8.5). Finally, the peptide of interest (4 mg, 1 mM in PBS, pH 8.5) was introduced, followed by PBS (pH 8.5) until the end of the measurement.

Supported lipid bilayers composed of POPE:POPG (3:1 mol:mol) were formed using the solvent-assisted lipid bilayer method, as described by Tabaei et al. (23) The sensor surface was primed with 3 mM CaCl2 for 5 min, followed by isopropyl alcohol (IPA) for 15 min. Lipids dissolved in IPA (0.5 mg/mL, 1 mL total volume) were then added. After lipid deposition, 3 mM CaCl2 and 150 mM NaCl were flushed over the sensor. A 15-min wash with PBS (pH 7.5) was performed to establish a stable baseline before peptide addition. The peptide of interest (4 mg, 1 mM in PBS, pH 7.5) was then introduced, followed by PBS (pH 7.5) until the end of the measurement.

### Circular dichroism spectroscopy

Circular dichroism spectra were recorded on a Chirascan V100 (Applied Photophysics Limited, UK) at 37°C using a 1-mm-pathlength quartz cuvette (Hellma Analytics). Spectra were acquired for 20 μM of peptide in PBS only (pH 7.5), PBS containing 0.25 mM LUVs, and PBS containing 1 mM SUVs. LUVs were prepared from lipid films (16:0-18:1 PE and 16:0-18:1 PG at molar ratio 3:1, Avanti), which were formed by the evaporation of chloroform and 3 h of vacuum drying. After drying, the lipids were dissolved in PBS (pH 7.5), followed by 10 freeze-thaw cycles (each consisting of 90 s in dry-ice ethanol bath followed by 30 s in 50° water bath). The resulting lipid suspension was extruded 30 times through a polycarbonate membrane filter with 100 nm pore size. The lipid films for SUVs were prepared the same way as for LUVs. After dissolving the lipid film in 1mL PBS (pH 7.5) the SUVs were created using the same method as previously described for QCM (10 min, 10-s pulses, 60% amplitude, on ice). Spectra were acquired at 1-nm intervals, with five accumulations, 1-nm data pitch, 1-nm bandwidth and 100-nm/min scan speed, within the wavelength range of 195–260 nm. Spectra were background-subtracted and converted to the mean residue molar ellipticity (in deg · cm^2^ · dmol^−1^ · residue^−1^) unit.

## Results

We calculated the PMFs of amino acid side chain analogs at POPE:POPG and POPC membranes. We selected POPE:POPG membrane as a simplified mimic of the bacterial plasma membrane whereas pure POPC membrane is a single-lipid mimic of the plasma membrane of mammalian cells. To validate the methodology, we chose very simple models for plasma membranes, using which we searched for peptide sequences with selective affinity toward different lipid headgroups.

**Figure 3:**
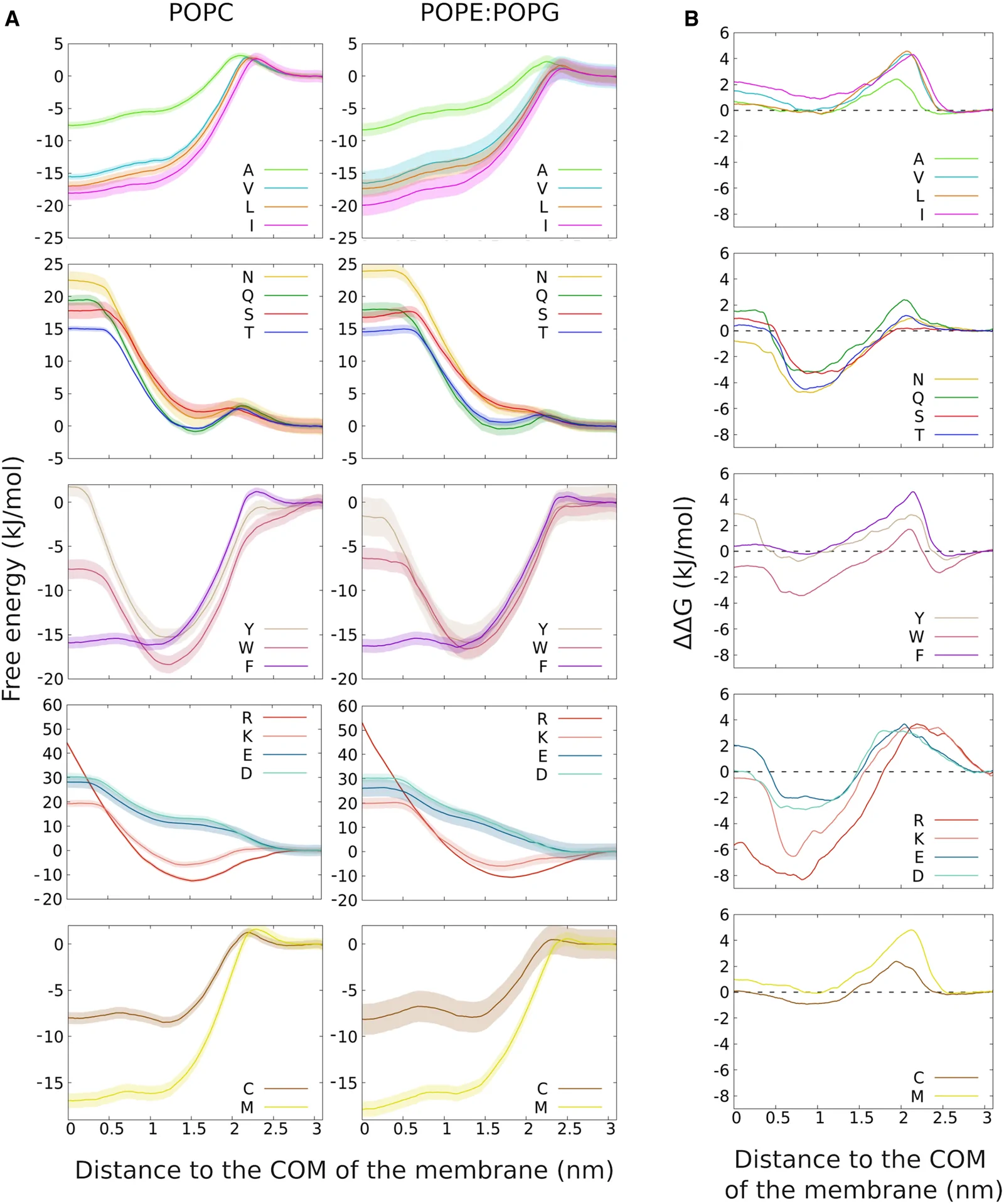
PMFs of side chains. (*A*) PMFs in POPE:POPG and POPC membranes. (*B*) Energy difference (ΔΔ*G* = Δ*G*_*POPC*_ − Δ*G*_*POPE:POPG*_) between the membranes for each side chain. The higher the ΔΔ*G* value is, the more selective toward bacterial membrane. Error bars were calculated using the bootstrapping method implemented in WHAM.

The PMFs are shown in Fig. 3 *A*, and the energy difference between POPC and POPE:POPG membranes is shown in Fig. 3 *B*. Importantly, there are few residues that have a preference for bacterial membranes, especially in the headgroup region (between 1.5 and 2.5 nm). Ile, Val, Leu, Phe, and Met are the ones with highest ΔΔ*G* and also the ones with almost no preference for POPC (small or no regions with ΔΔ*G* < 0). Charged residues have also a high preference for POPE:POPG headgroup region, but they also have the opposite behavior in the tail region, with especially low ΔΔ*G* values for positively charged residues.

The final PMFs of charged amino acids were obtained by doing the Boltzmann average between the charged and neutral states of the side chains (see Fig. S3). The neutral side chain profiles are shifted by the free energy it would require to neutralize the residue in water (24). The averaging then provides the profile that takes into account possible protonation/deprotonation states along the side chain insertion into the membrane.

During the pulling of some of the side chains inside the membranes, we observed water defects. The defects are created mainly by the charged side chain dragging of water molecules inside the membrane and are in agreement with a previous report (24). However, most of the charged side chains are more likely to be in their neutral form, which does not create a water defect, once they enter the lipid tail region, as shown in Fig. S3. The only exception is arginine, for which the charged state seems more favorable than neutral state even at the center of the membrane. Therefore, it is possible that the free energy at the center of the membrane of charged arginine is not well estimated because of the presence of the water defect. Nevertheless, the middle of the membrane remains highly unfavorable for all charged residues.

In addition to the single side chain analogs, we also calculated the PMFs of two strongly interacting side chains. We focused on salt bridges and aromatic residues. As expected, all combinations lead to the same behavior with the lower free energy for the interacting pair compared with the summed profiles of individual side chains; see Fig. S4. In the case of aromatics, the result depends on the aromatic side chain involved. The biggest changes are observed in interaction between two tryptophans, whose energy minimum is much lower when we sum the two side chains than what we obtain when simulating them together, and in the interaction between two tyrosines, which have a lower energy barrier at the membrane center when simulated together. For a complete display of the PMFs of salt bridges and aromatics, see Figs. S4, S5, and S6.

### Sequence prediction

The main aim of the work presented here is to predict peptide sequences that would selectively bind to membranes composed of POPE:POPG lipids (used as model for bacterial plasma membrane). To do this, we used the PMFs of amino acid side chains that we obtained from MD simulations to estimate the free energy difference of 20 amino acid long peptides for POPC and POPE:POPG membranes. We will refer to this energy estimation from the side chain contributions as the implicit model. We used a genetic algorithm to select sequences with the highest energy preference for the POPE:POPG membrane (larger ΔΔ*G*); for details, see the materials and methods section. Since we are interested in sequences that adsorb to the membrane, the location of the energy minimum should be between 1.5 and 2.5 nm, especially in POPE:POPG membrane. The energy minimum in POPC should be either in the same adsorption region or in the water phase (meaning that it would not bind to the membrane at all). We also prefer helical sequences because we assumed alpha helical secondary structure in the energy calculation of the genetic algorithm. Examples of selected sequences can be found in Table 1.

**Table 1:** Table containing the sequences with highest ΔΔ*G* obtained from the genetic algorithm, the locations of the energy minimum in each membrane and their predicted helicity

Sequence	ΔΔ*G*	Min. PC (nm)	Min. PE:PG (nm)	Helicity
FIKDEIDKADEEMWERYDKE	45.6047	1.5	1.9	—-HHHHHHHHHHHHH—
QELIMDQMEEMDDKMEKMEE	44.998	3.9	1.9	-HHHHHHHHHHHHHHHHH–
KEIIEDIYDDLEDKMDFMEE	50.7359	3.9	1.9	-HHHHHHHHHHHHHHHHH–
ELFRDMEEEFMDDFEDKMER	50.8394	3.8	1.9	–HHHHHHHHHHHHHHHH–

**Figure 4:**
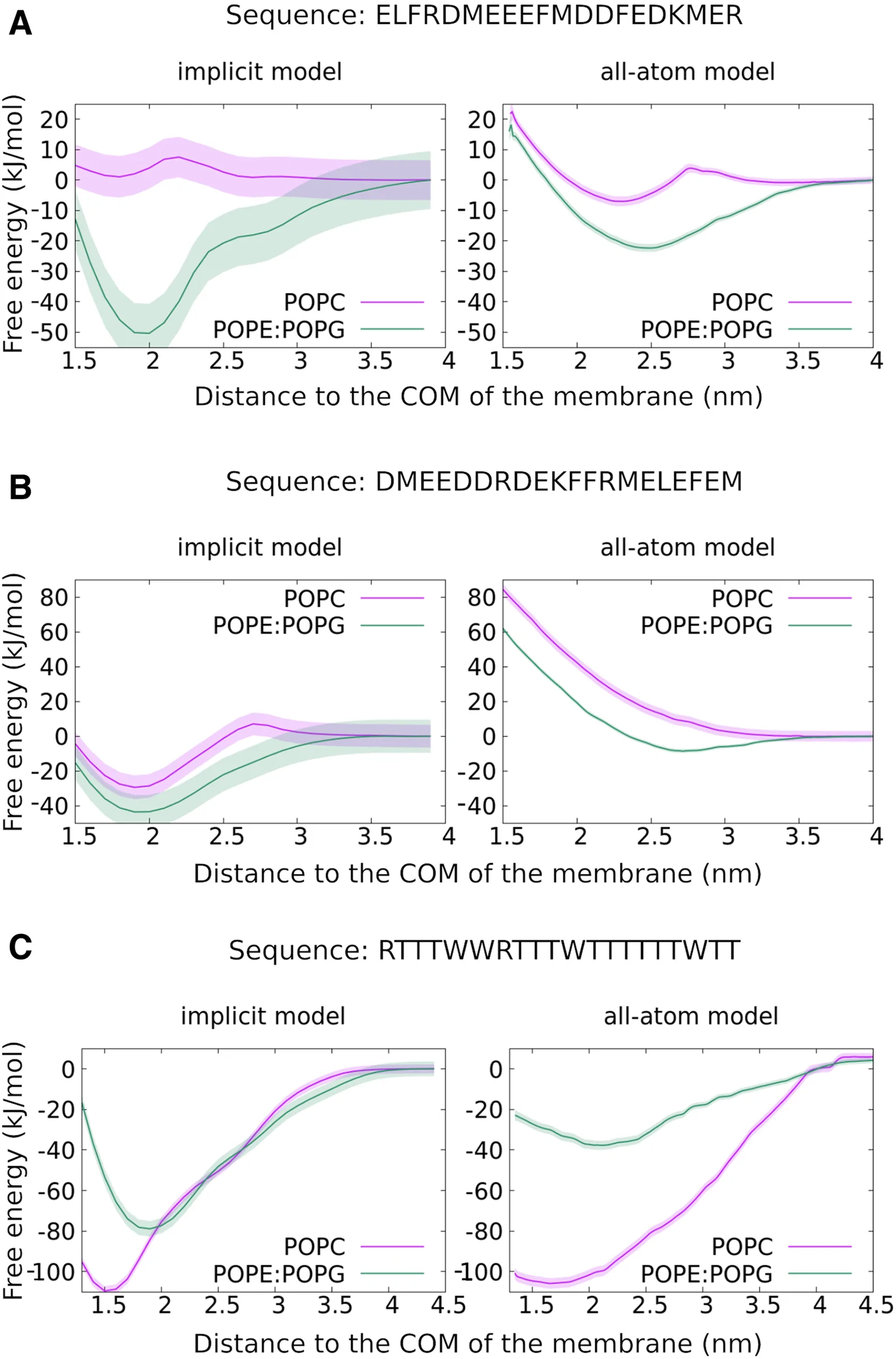
Comparison of the PMFs predicted by the implicit model (*left*) and calculated from all-atom model of the whole peptide (*right*). X-axis is the distance between the center of mass of the peptide and the center of mass of the membrane. (*A*) PMFs of one of the most POPE:POPG-selective peptides obtained from the genetic algorithm. (*B*) PMFs of a peptide with the same amino acid composition as (*A*) but with scrambled sequence. (*C*) PMFs of a POPC-selective peptide. The error bars in the all-atom PMFs are calculated form the bootstrapping method implemented in WHAM, and the error bars from the implicit model are calculated using the additive formula for error propagation.

To confirm the results obtained from the genetic algorithm and implicit membrane model, we calculated the PMFs in POPC and POPE:POPG membranes of one of the peptides (sequence: ELFRDMEEEFMDDFEDKMER) using all-atom simulations with US. The results from US (see Fig. 4 *A*) show that the sequence is indeed selective toward bacterial membrane, as it has lower energy minimum for POPE:POPG than for POPC. Nevertheless, there are some differences between the energy obtained from US of the whole peptide and the one predicted with the implicit model. The first difference is the value of the ΔΔ*G* between the minimum of each membrane. Although the implicit model predicted a ΔΔ*G* of around 50 kJ/mol, the result from all-atom simulations is 20 kJ/mol. The second difference is regarding the position of the energy minimum, which is shifted 0.5 nm toward the membrane center in the implicit model.

To ensure that the peptide selectivity depended on the sequence and not only on the amino acid composition, we also evaluated the energies of peptides with same composition as ELFRDMEEEFMDDFEDKMER but scrambled sequences. Most of the scrambled sequences had ΔΔ*G* values below 30 kJ/mol, lower than the original value of 50 kJ/mol. We selected one of the scrambled sequences (DMEEDDRDEKFFRMELEFEM) to validate the PMF with all-atom simulations. The result of the scrambled sequence can be observed in Fig. 4 *B*. The energy minimum is also shifted in the all-atom results compared with the implicit model, and the ΔΔ*G* is smaller (implicit model 12.5 kJ/mol, whereas all-atom 7 kJ/mol).

We also tested a POPC-selective sequence (RTTTWWRTTTWTTTTTTWTT) and validated the PMF with all-atom simulations; see Fig. 4 *C*. The PMF from all-atom simulations agrees with the implicit model in the peptide preference to POPC membrane compared with POPE:POPG. However, as it occurred in the previous sequences, there are some differences between the implicit model PMF and the one obtained from all-atom. The energy minimum, in this case, is less shifted than in the previous sequences, only 0.1–0.2 nm toward the membrane center in the implicit model compared with all-atom. The second difference in the value of the ΔΔ*G*, which is smaller in the implicit model (−30 kJ/mol) than the one obtained from all-atom simulations (−68 kJ/mol).

### Backbone effects

The differences between the implicit and all-atom models suggest that there may be an additional contribution apart from the side chain PMFs. This could be due to cooperative effects between nearby side chains, similar to the effect observed for salt bridge or aromatic interactions, or because of the effect of the backbone volume when introducing a large alpha helix into the membrane.

**Figure 5:**
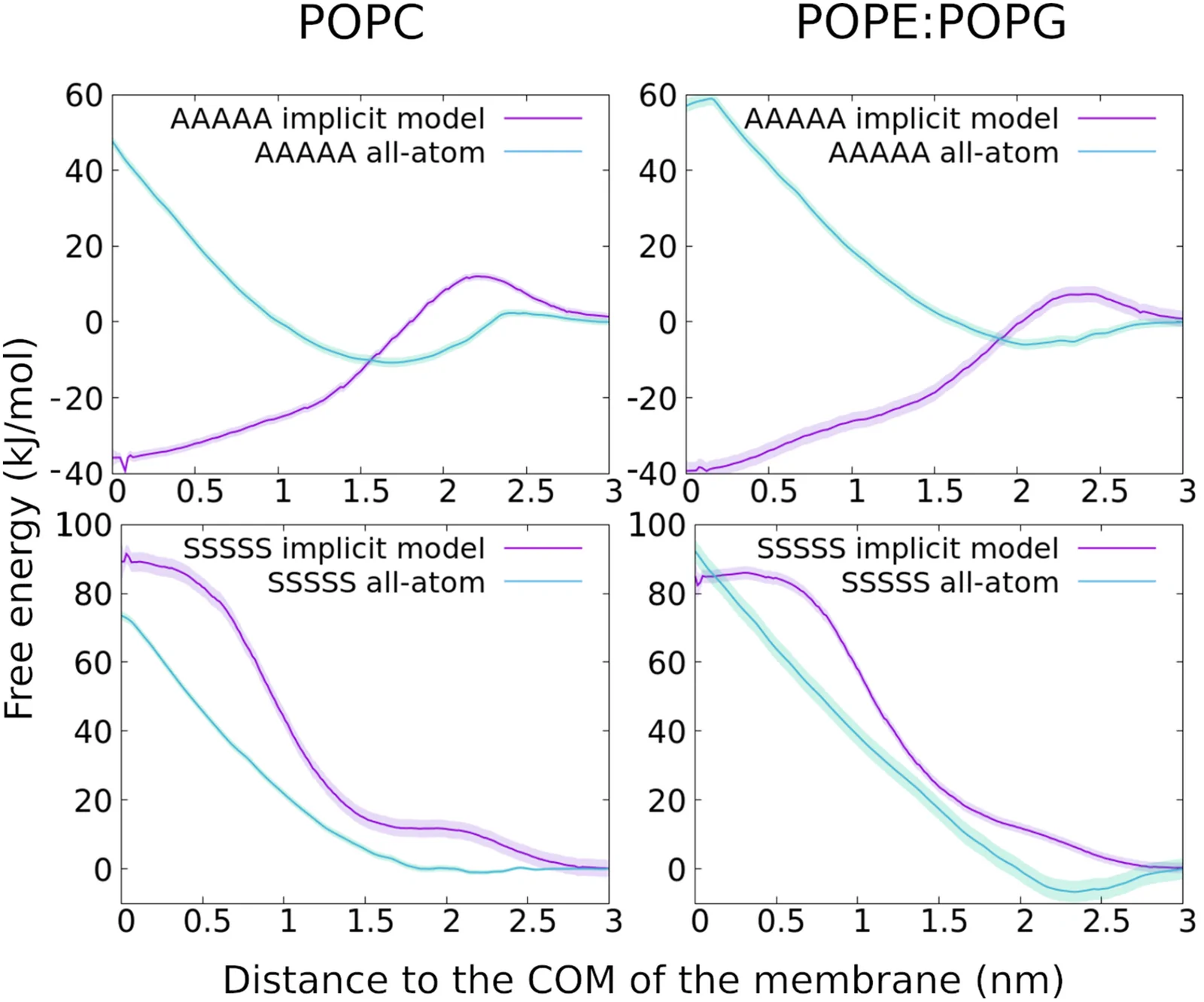
Comparison of PMFs of alanine and serine pentapeptides obtained from the implicit model and obtained from all-atom simulations of the whole pentapeptide. The error bars (*colored area*) in the all-atom PMFs are calculated from the bootstrapping method implemented in WHAM, and the error bars from the implicit model are calculated using the additive formula for error propagation.

We investigated the possible effect of the peptide backbone using pentapeptides (composed of alanine and serine) and compared it with the PMF calculated with the implicit model; see Fig. 5. For the alanine peptide, the PMF from the implicit model has the minimum at the center of the membrane, but the PMF from all-atom shows that the minimum would be at 2 nm from the membrane center, with a high energy barrier at the center of the membrane that is not captured by the implicit model. For the serine peptide, the implicit and the all-atom model results show a similar trend, although the energy from the implicit model has higher values.

**Figure 6:**
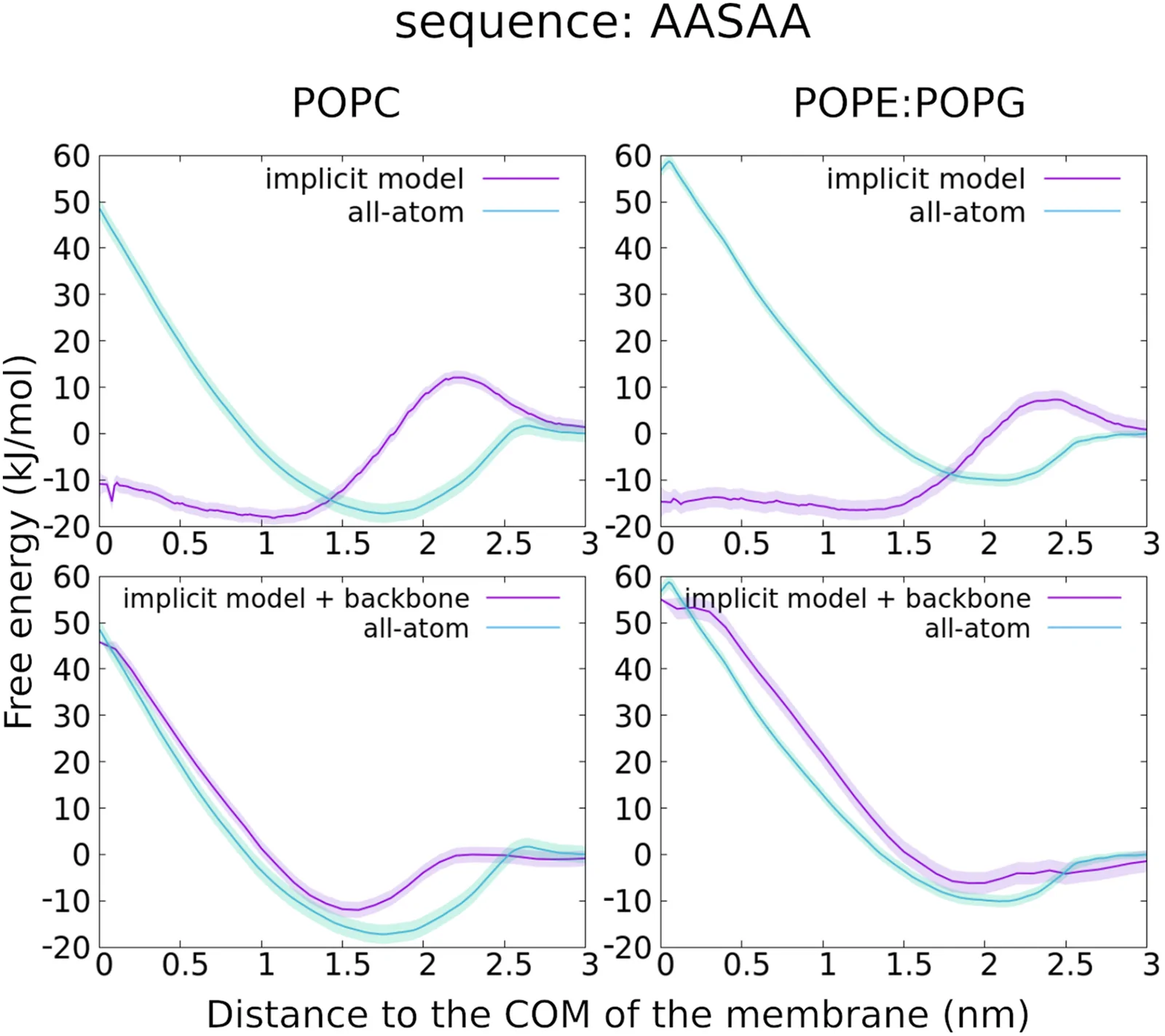
PMFs of AASAA peptide obtained from the implicit model without adding the backbone contribution and adding the backbone contribution compared with the PMF obtained from all-atom simulations of the whole peptide. The error bars (*colored area*) in the all-atom PMFs are calculated from the bootstrapping method implemented in WHAM, and the error bars from the implicit model are calculated using the additive formula for error propagation.

We calculated what would be the backbone contribution for both pentapeptides by subtracting the side chain energy contributions from the all-atom PMFs. We obtained different backbone contributions (see Fig. S7) depending on the side chain composition of the peptide. Note that there are only very small differences for different membranes. To assess if the addition of the backbone contributions would improve the free energy prediction, we also calculated the peptide with sequence AASAA (Fig. 6). The PMFs from the implicit model including backbone contribution improved the agreement with the all-atom PMF. The backbone contribution also improved the location of the free energy minimum in both membranes. The ΔΔG between the membranes from the implicit model with backbone contribution was near −6 kJ/mol, very close to the value obtained from the all-atom model, which was −7 kJ/mol. In comparison, the ΔΔ*G* obtained from the implicit model without the backbone effect was of −2.3 kJ/mol. Therefore, the estimate of the ΔΔ*G* also improved with the addition of the backbone effect.

As seen in Fig. 5, the effect of the backbone is more significant in peptides with high alanine content, since it modifies the overall shape of the free energy profile and therefore the location of the energy minimum. After further testing with leucine pentapeptide, we obtained similar results to alanine, and therefore, we expect that hydrophobic residues have similar backbone effect.

### Experiments

We selected several peptides with selectivity toward POPE:POPG membrane, according to the implicit model, to test them experimentally and measure the differential binding to both model membranes: POPC and POPE:POPG. The peptides were obtained from the final cycles of the genetic algorithm evolution, and we selected peptides with varied sequences. Despite the sequence variation, the selection of peptides was not alpha helical, so we also designed one peptide (NKL) with a secondary amphiphilic character, which is likely to become helical once adsorbed to the membrane. Because we assume alpha helical structure of the peptides in the implicit model, the binding of the peptide to the membranes should more closely capture the predicted membrane selectivity. As controls, we also tested one peptide with predicted stronger affinity to POPC membrane, PC4, and one peptide without selectivity (only small calculated ΔΔG) between membranes, SC11. More peptides were ordered, but they turned out not to be soluble, therefore not usable for experiments. The tested peptides are shown in Table 2. Note that the ΔΔ*G* of NKL peptide is calculated including the correction of the backbone contribution. We added as an approximation the backbone contribution calculated for alanine to all hydrophobic residues, the backbone contribution calculated for serine to hydrophilic residues, the backbone calculated from tryptophan to aromatic residues, and the backbone from lysine to charged residues.

**Table 2:** Table shows the peptides that were tested experimentally

Name	Sequence	ΔΔ*G*
NR0	REERREEREEKRRRRDEERD	46
NR1	EDRREERRERREERREEREK	38
NR2	EDRREEERERKEERMEEKRK	42
NR3	KRREEMEERRREEKERRDDE	41
NB1	FIKDEIDKADEEMWERYDKE	43
23SB2	ERSESREEMWDEMAEEREEW	32
23SB3	REDKSEMREEKSEMREDRME	38
PE4	MDEDAKDKEEVVEEMMEDRD	13
PE6	YKDKYDKLQKDVKDKLDKDD	40
PE7	EKDKKVKVKKDKKDMRDEKD	40
PC4	ARRARWRAAARWARRRWWWR	−31
SC11	ERKEEMSSDEREDEKMRREM	12
NKL	SLKKLLKKLNSLLNNLKSSL	12^∗^
PE9	KAKRVAWQVYQAAKKVKQVA	16^∗^
PE12	QKKQIKKKINNIKKQIKQKI	25^∗^

First column shows the identification name, second column is the amino acid sequence, and third column is the value of the ΔΔ G predicted by the implicit model. Positive values of the ΔΔ G mean there is selectivity toward POPE:POPG membrane, whereas a negative value means the peptide is selective toward POPC membrane.^∗^ΔΔ*G* was obtained with correction for backbone contribution.

**Figure 7:**
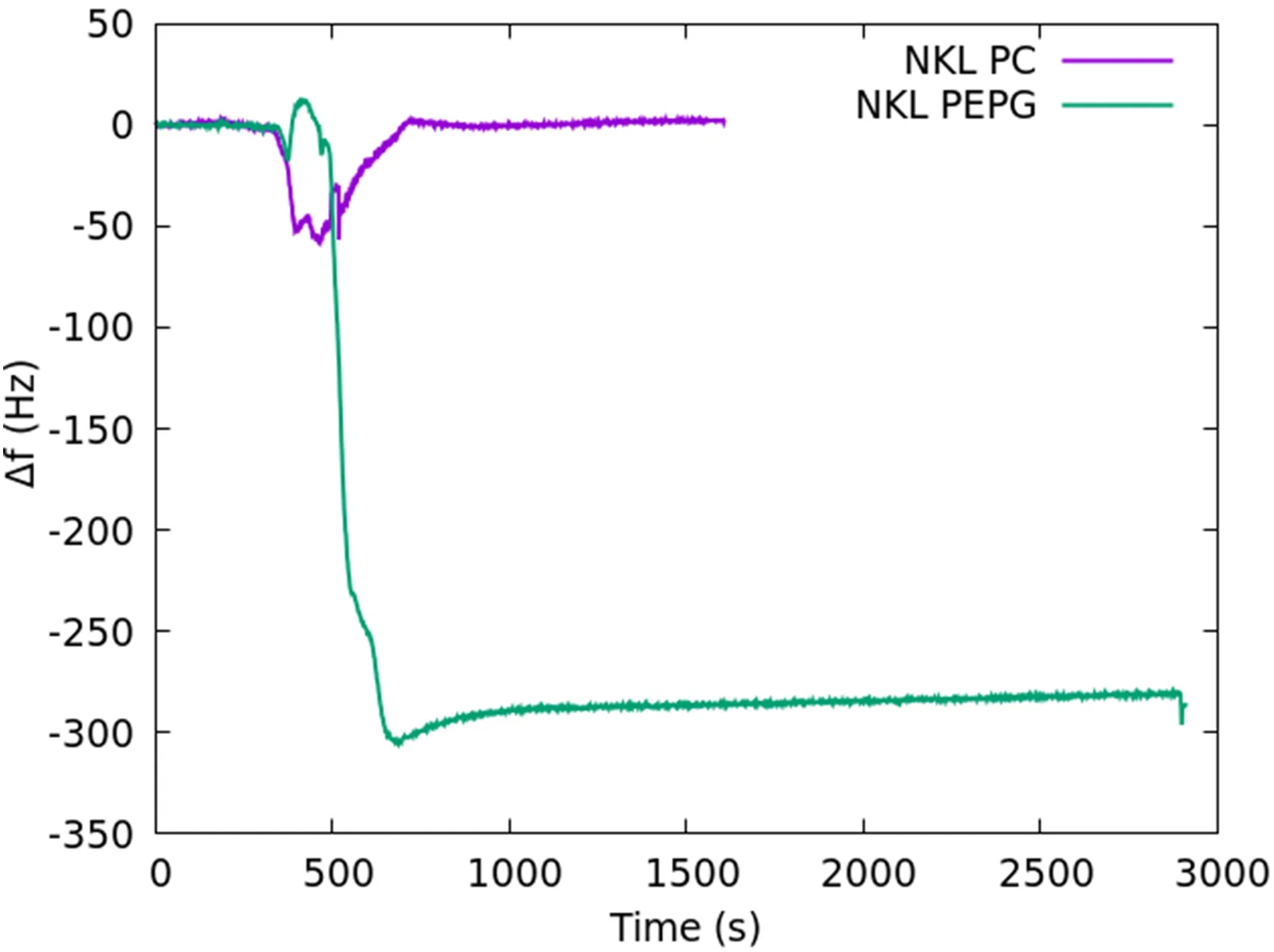
QCM result of NKL peptide, which was designed with a hydrophobic face on helical structure. Peptide was added at 200 s. The decrease in frequency captures the amount of peptide adsorbed to the bilayers. The data are shown as the average of four replicas, and only the fifth overtone has been considered for simplicity. Both x and y axis are shifted so that 0 represents the stable bilayer without peptide. The selectivity is much larger in this peptide, probably due to its secondary structure, which was assumed in the implicit model.

As shown in Fig. S13, all peptides are mostly unstructured in PBS solution, and most of them are also unstructured in the presence of LUVs. The only exception is NKL peptide, which has alpha helical conformation in the presence of LUVs, and there is also a hint of helical structure for peptide PC4 in presence of LUVs, but it is much less clear/structured. This means that most QCM results are hard to compare with the implicit model calculation with the exception of NKL peptide. And, as observed in Fig. 7, NKL peptide shows great binding selectivity toward POPE:POPG membrane, as was obtained from the implicit model.

Although the rest of the peptides do not form helical structures, we also tested them for POPE:POPG selectivity. In many cases, there is a small selectivity for POPE:POPG membrane, see Fig. S12. However, as can be expected due to the lack of helicity of these peptides, there are also QCM results that contradict the predictions of the implicit model, as shown in Figs. S10 and S11.

Finally, we designed additional peptides with hydrophobic face, as was done for NKL peptide, with the aim of obtaining additional alpha helical peptides that we could properly experimentally compare with the implicit model. The peptides we obtained are PE9 and PE12 (see Table 2 for sequences and ΔΔ*G* values). Unfortunately, these peptides also resulted to be unstructured both in PBS solution and in the presence of LUVs or SUVs; see Fig. S16. Still, we obtained their preferential binding through QCM experiments. As seen in Fig. S17, there is a small difference in the selectivity of both peptides to our model membranes. These results further highlight the main drawback of our method, which is the need for helical structure leading to the scarcity of experimental comparisons.

## Discussion

Peptide selectivity toward specific membrane compositions can be useful for targeting drug delivery systems to specific cell types. Here, we focused on alpha helical peptides that would have higher affinity toward a mimic of bacterial membranes compared with mammalian plasma membranes. Although the selected membrane models are simplified, designing peptides with high affinity for these lipid headgroups is a critical first step toward developing peptides that selectively bind to more complex biological membranes. Such selectivity toward bacterial surfaces is expected to locally enhance peptide concentration and increase antimicrobial activity, regardless of the specific mechanism of action.

There have been many previous attempts to increase peptide selectivity toward specific membranes (25,26,27,28,29,30). However, the selectivity mentioned in these studies corresponds to a higher/lower activity of the peptide, and there is little information about how differences in the binding affinity between the membranes is contributing to the overall selectivity. There are few studies in which the binding selectivity of peptides is the main focus of the study (31,32), but we still lack the knowledge on how to improve the selective binding of peptides to certain membrane compositions. Therefore, further investigation about peptide binding selectivity would be beneficial to improve the therapeutic efficacy of membrane-active peptides. Peptides that would also benefit from this knowledge in a great degree are peptides used for targeting or tracking of lipids (33) for which membrane activity is not necessary.

The work presented here aims to fill this gap by developing an implicit membrane model by which we can easily test the selective binding of helical peptides to different membrane compositions. More specifically, we test preferential binding to different lipid headgroup compositions using membranes with the same lipid tails. We used the free energy contributions of amino acid side chains in the membranes of interest to predict the free energy differences of a peptide binding to those membranes and therefore its binding selectivity.

Our results on POPC membrane correlate well with the hydrophobicity scales (24,34,35), as shown in Fig. S8, and with previous simulations results by MacCallum (24) and Marx (36) (see Fig. S9). Hydrophobicity scales provide the opportunity to calculate whole peptide partitioning free-energies from the contribution of its constituent residues (37), as well as to build realistic models for protein design (38). We also adopted this simplified approach and calculated peptide affinities from its constituent amino acids and used a genetic algorithm to optimize the sequences. This strategy allowed us to discover a wide variety of peptides with binding selectivity toward POPE:POPG membranes (Table S1). Note that for salt bridges and aromatic stacking residues, we implemented a correction for such clearly nonadditive behavior. Also, other residues may exhibit nonadditive behavior, such as multiple charged residues sharing many water molecules in the hydrophobic part of membrane. However, these states generally induce significant membrane deformation, which lies outside the scope of our model. Such deformations are energetically demanding and are thus not expected in selectively adsorbing soluble peptides. For other residues, we assumed that the nonadditive effect will be similar on both membranes and thus would not affect the selectivity dramatically.

In addition, we assumed that the peptide sequences adopt an alpha helical secondary structure within our implicit membrane model. We acknowledge that peptides are highly dynamic and often unstructured in solution. However, many peptides adopt a defined secondary structure upon contact with a membrane, and this structure is likely to be similar across different membranes. Therefore, the folding energy is expected to be comparable, and thus the membrane selectivity could be conserved also for nonhelical peptides as observed in some of our experiments. Note that we also used the alpha helical secondary structure in our validation all-atom simulations of peptide selectivity. Without additional testing, we thus recommend filtering the identified selective sequences based on their predicted helicity.

However, as seen from the results of circular dichroism, even though our peptides were predicted to be alpha helical, most of them were actually unstructured in PBS and in the presence of lipid vesicles. This makes a difficult comparison between the prediction from the implicit model (where the peptides are assumed to be alpha helical) and the experiments. The only alpha helical peptide we obtained was NKL, which we designed to have an amphipathic character, forcing it to have a hydrophobic face. This peptide turned out to have strong selectivity toward PE:PG lipids, matching the implicit model prediction. Therefore, it might be better to modify an existing alpha helical peptide rather than design new selective peptides de novo.

The selectivity of few obtained sequences was validated with all-atom simulations using US. The main differences seem to originate from the lack of a backbone contribution. As seen in Fig. 6, the lack of backbone effect can result in large discrepancies between our model and the verification simulations. Side-chain correlations can also play a role, and we calculated those for salt bridges and aromatic residues; see Figs. S4, S5, and S6. There are important differences between membranes for salt bridges, whereas for the aromatic residues the correlations are much smaller when comparing between both membranes; see Figs. S14 and S15. Therefore, we anticipate that differences between membranes for correlations of other side chains are even smaller and thus are less important for the selectivity. The effect of the backbone contribution appears to be more prominent for hydrophobic side chains than it is for polar side chains, and the addition of the backbone contribution to an AASAA peptide improved the agreement with the all-atom PMFs, as well as predicted a ΔΔ*G* closer to the value obtained from all-atom. The differences in the backbone contribution depending on the side chain complicate the model derivation. The difference might originate from the various environments of the backbone, i.e., interaction with different atoms of side chains and/or modified availability of the surrounding solvent caused by different side chains. We calculated a backbone contribution for each type of side chain (hydrophobic, hydrophilic, aromatic, and charged), and we tested it on the affinity of NKL peptide, which at least qualitatively matches the experimental results. The use of pentapeptides for the calculation of backbone contribution might not be ideal due to the available hydrogen bonds from a large part of backbone in a pentapeptide. However, longer polypeptides resulted in large membrane deformations. Therefore, future efforts might address backbone contribution differently.

Despite the differences in PMFs for individual membranes, the membrane selectivity of peptides generated with implicit model without backbone contribution agreed well with all-atom simulations of the peptides. Therefore, using the implicit model, we were able to find peptides with high preference toward POPE:POPG membranes, as well as peptides with preference toward PC membranes. Future improvements such as the already discussed addition of backbone effects or additional side chain correlations could make the predictions much more accurate.

We also validated our results experimentally using QCM to measure the binding of the peptides to POPC or POPE:POPG membranes. We found that for the only peptide that adopted alpha helical secondary structure, the binding selectivity matched the prediction from the implicit model. The rest of the peptides did not adopt alpha helical structure, despite the secondary structure prediction from Jpred. For a more successful selection of alpha helical peptides, it will be necessary to either develop a more suitable model for secondary structure prediction or to follow the strategy of designing secondary amphipathic peptides that are more likely to become alpha helical upon adsorption to the membrane, as we did for NKL peptide.

## Conclusion

In this paper, we have developed an implicit membrane model to efficiently determine alpha helical preferential adsorption of alpha helical peptides to POPE:POPG membranes (that mimic bacterial membranes) compared with POPC membranes (mimicking mammalian membranes). The preferential adsorption was verified using all-atom simulations calculating the free energy difference between membranes and through QCM experiments. Because the model assumes a helical peptide structure, designing novel sequences needs to be coupled with robust secondary structure prediction. Alternatively, the model could be used to modify existing helical motifs. The discovery of selective peptides can have applications in bacteria targeting drug delivery or design of antimicrobial peptides with reduced toxicity.

## Data and code availability

All the necessary files to reproduce our data, including topologies, force field parameters, and input configurations, are openly available on Zenodo at https://doi.org/10.5281/zenodo.14724572.

## Acknowledgments

The work was supported by the 10.13039/501100000781European Research Council under the 10.13039/501100007601European Union’s Horizon 2020 research and innovation program (grant agreement no. 101001470) and the project National Institute of Virology and Bacteriology (Program EXCELES, ID project no. LX22NPO5103)—Funded by the European Union — Next Generation EU. Computational resources were provided by the CESNET, CERIT Scientific Cloud, and IT4 Innovations National Supercomputing Center by MEYS CR through the e-INFRA CZ (ID: 90254).

## Author contributions

S.B. carried out all the simulations and analyses. E.S., M.D., and V.R. performed all experiments. R.V. supervised and designed the research. All authors contributed to the discussion, writing, and revision of the manuscript.

## Declaration of interests

The authors declare no competing interests.

## Declaration of generative AI and AI-assisted technologies in the writing process

The authors acknowledge the usage of Grammarly and ChatGPT for improving the readability and language of the manuscript.

## Supporting material

Document S1. Figures S1–S17 and Table S1

Document S2. Article plus supporting material
