# Supplementary material for: Implicit membrane for helical peptide selectivity toward bacterial membranes
Source: Biophys J. 2026 Apr 6;125(9):2164–74. doi: 10.1016/j.bpj.2026.03.059 (PMC13351856; doi:10.1016/j.bpj.2026.03.059)
Supplement: Document S1. Figures S1–S17 and Table S1 [file mmc1.pdf]

Biophysical Journal, Volume *125*

Supplemental information

Implicit membrane for helical peptide selectivity toward bacterial membranes

Sofía Blasco, Erin Spearing, Martina Drabinová, Vendula Rašková, and Robert Vácha

Supporting Information

Implicit Membrane for Peptide Selectivity Towards Bacterial Membranes

Sofía Blasco,[†,‡] Erin Spearing,[†,‡] Martina Drabinová,[†,‡] Vendula Rašková,[†,‡] and Robert Vácha[*,†,‡,¶]

† CEITEC – Central European Institute of Technology, Masaryk University, Kamenice 753/5, 625 00 Brno, Czech Republic
‡ National Centre for Biomolecular Research, Faculty of Science, Masaryk University, Kamenice 753/5, 625 00 Brno, Czech Republic
¶ Department of Condensed Matter Physics, Faculty of Science, Masaryk University, Kotlářská 267/2, 611 37 Brno, Czech Republic
* E-mail: robert.vacha@muni.cz

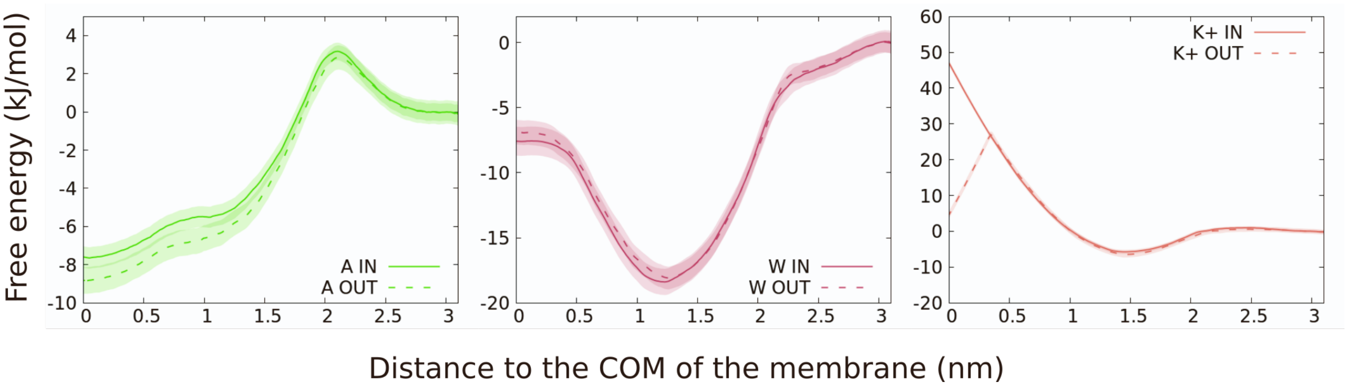

Figure S1: Comparison of PMFs obtained from opposite pulling directions, from the water phase to the center of the membrane (IN) or from the center of the membrane to the water phase (OUT).

Sequence	$\Delta\Delta G$	min. PC	min. PE:PG
DRKDRKDMKDQKDDYLDDFY	50.2588	3.3	2
DYKDMYDDWMNVDRDDLDDY	50.573	3.9	2
DKKDLKDDKWDVYVDVKDDK	53.7061	3.9	2
DMDDYDFDLKDLMDDFADKY	42.8681	1.3	1.9
DMDDMDFDMRDRKDVMYDDK	47.4105	3.9	2
DKDWASYDFDDLFDDLLDKY	38.5642	1.3	1.9
RDRKDRKLDRDDKDWKDYKD	47.9877	3.2	2.1
YKDKYDKLQKDVKDKLDKDD	41.7766	2.3	2
DRKDKRDRRDNQLDDMDDKL	49.9361	3.9	2.1
QLDDKLDLMDDFYDDLDDLF	42.9925	1.3	1.9
DMDDLLDDFLDLYDDLMDDL	44.5281	1.2	1.9
AYDDLLDDLLDLLDDLLDDK	41.0371	1.2	1.9
DRDDKDMVMKDKKDMYDDKY	42.631	1.4	2
DKYYFDLDDMLDDKQDKLDK	39.4123	1.3	1.9

KKDKKDKLYKDWKDMLDDDQ	42.1638	1.4	2
DLKDLLDDLLDLKDDLLDDL	42.8166	1.2	1.9
KRDKQQKDKKDQKDDKMDKL	48.8715	3.3	2
KKDKKDRKDKQLKDKLDKKD	51.3065	3.9	2
DDKQDWKQYAKRDKKDMRDD	47.097	3.9	2
KDQYDDKLDRLWKDYKDDKD	51.5983	3.9	2.1
YKDQLQDVKMDQKDDLDDKL	44.2407	3.9	1.9
RDDRQDNVVDDLLDDYVDKY	49.3398	3.1	2
DKKDKKQVKKDVKDDLLDDF	50.916	3.9	1.9
KDYKDLLLDDLLDLLDDLDD	42.5132	1.2	1.9
DLKDKKSQKKDLKDDLLDDL	48.496	3.9	1.9
DKKDKKQQKKDALDDMMDDF	48.7452	3.9	1.9
KDDKLDKKDKKAQMKDDYYD	45.915	3.9	1.9
LLDDKDDYLDWMQDDYMDDF	47.5617	3.9	1.9
DKDDMANVKKDLLDDYMDDY	50.5985	3.9	1.9
DDRDWMDYMDDDLDDLMDKF	52.809	3.9	2
YYDDLLDLLDDLLDDKMDKD	44.4543	1.3	1.9
MKDDKLDLYDDWLDMLDDDL	44.3279	1.3	1.9
KDLLDDLLDLLDDLLDDKLD	43.4977	1.2	1.9
MDDKQDLFDDLLDDLDDYYL	42.9614	2.9	1.9
DDYLDDLLDVLDDLYVDRKD	41.5901	1.3	1.9
DDMDDQMYDLWCDMRDMKDD	49.5838	3.9	2
RDKKDVYDDWADKKQNMVDD	45.2607	3.9	2
DDKLDKLLKDKKDKKDSRQK	47.9542	3.3	2
YKDKKDQLVDVLDDLLDDLD	40.7271	1.3	1.9
RDQLDDLQFDLFNDKKDKKD	46.2322	3.7	2
DMRDRDSNAKDDKVDMMDDL	45.602	3.9	2
DKRLKKDMLLDDLQDMDDDM	36.5878	1.3	1.9
QYQKDKLDKLDKVLKDDRDD	44.1831	3.9	2
SKDDKDSQFKDMLVDLKDKQ	39.5755	3.9	1.9
KVQYDDKQSKFKNDLDDKLD	39.6025	3.9	1.9
KKDSVQYDQWDDKVDALQDL	31.4544	1.4	1.9
VYDDMLDDYLDDLMDDMDDM	43.3214	1.2	1.9
DYYDLLDDMLDDLMDDMDDL	44.0653	1.2	1.9
QLLDDWDDYMDDMMDDMDDM	54.3055	3.9	2
QFKDDKDDLMDDLMDLYDDL	50.2433	3.9	1.9
LYKDDLDDLLDVKDDLLDDL	55.9328	3.9	1.9
AYKDLKDDLLDVKMDQDDDM	46.9834	3.9	1.9
DMKDLKQDFLDDLDDLLDDM	53.7392	3.9	1.9
DYKDALMDFLDDLDDLLDDK	51.4078	3.9	1.9
KDMKDDKMDMLDDLMDMLDD	54.4001	3.9	1.9
MDMKDDLQDLLDDKMDKKDR	54.5823	3.4	2
DDVMVDYKDWKDDKLDKKDR	47.5345	3.8	2
DYKLDLDDKLDSCWSDYKDK	46.5317	3.9	2
DFMDDFLDMKDDMMDDLDDM	54.5069	3.9	2
DLFDDMLDMFDDQLDDKLDM	43.3844	1.2	1.9
KFDYDDWFDDKQYLKDDKMD	49.4952	3.9	2
MDMYDDKDDRMQRDRDDRDK	47.0855	3.9	2.1
MDDDMDDLMVDLKDKYDKQY	39.694	1.3	1.9

WDLYDDKDDRLSRDRSDVKD	48.7363	3.9	2.1
MKDYKDDLVDVLDDLLDDLD	43.228	1.2	1.9
QYDYLDDLLDKLDQLLDDKD	48.7705	3.9	1.9
RDDKDMKVDLLDDLLDKQQK	38.7813	1.4	1.9
YDDKLDVLLDKKDSLLDDMD	50.316	3.9	1.9
WDVYNDDKDSKDKQLYDKWD	45.2922	3.3	2
YDDMMDYLDDYLMDLDDDMD	42.3888	1.2	1.9
LDDMDDLLDDKVDKKLSKRD	48.9091	3.9	2
RDLKDVYDDWADDLDDRMLS	40.6601	1.3	2
LKDDLDDLLDDLMDDFVDKL	43.4388	1.2	1.9
DKDMEDMVEMDMEMMDDEMD	59.7753	3.9	2
EMKEMVDDDMEEVVEMEDRK	60.986	3.9	2
EERDMMEKMDEDVVDMKDDM	57.8022	3.9	2
EDMMDEMDVVVEDMEDMKDK	58.5051	3.9	2
EMDDMMDDMMEEMDDREKRE	56.4236	3.9	2.1
MDDMMDDVEEVEMDKMDKKE	58.7071	3.9	2
DDMEEEMKEMVVDMKDMKDD	55.4618	3.9	2
DMEDMKDDVMEMVEDDEDMM	58.6208	3.9	2
MDKVEEDMEMVDDDAMDMMD	56.3878	3.9	1.9
AKDMMDDDVEEMMDMEDRKE	57.1043	3.9	2
EMMDMDDDMMEEADEMMKDK	57.3348	3.9	2
KDMEDMEDDVMDMMDDKEDR	57.8483	3.8	2.1
MDEMDDMVEEVMEEMDEMDK	59.2422	3.9	2
EMEDKMEEVMEEVEDMDDKM	60.3045	3.9	2
DMKDMMMDDEEMEEDKMDKM	57.1696	3.9	2
EVEMDKMDMDDMMDDVMDDM	53.7916	3.9	1.9
EMEDMDMDVMEMKEDEMEDM	59.4434	3.9	2
KDMMDDMVDEMEDMMEMMDE	40.2309	1.2	1.9
KEMKDMKDEMMEDMDEKMER	55.6536	3.9	2
EEMMDEKDDVMMDMMSEKRD	51.3588	3.9	2
VRDDKDEVMMDVEDVCEDYM	36.5856	1.3	1.9
MDVKEDMEDMMDEMMEDMME	41.8554	1.2	1.9
KEMKDDMEDMMDDMMVDMKD	41.7395	1.3	1.9
AKKDMKADMKMDEDDMMDEM	35.8762	1.3	1.9
VVDDMMDEKDDVMEDKVDKM	55.1434	3.9	1.9
EDMMDVKDDMMDMKADMMED	41.8937	1.3	1.9
RDVMDDMMEEKVEKMDVKEE	53.8914	3.9	2
EECVDMMDDMMNEKRDKKDK	39.485	1.4	2
EKDMKEDMMEDAMVDMKDKY	36.0784	1.3	1.9
KDVMDDKMDVMDDQAADMMD	38.9848	1.3	1.9
EDMMWDMMDEEDEKMDSKMK	40.434	2.7	1.9
DKDNMMRDERDVWADEKDKK	47.8766	3.9	2
KDDMDDKMVKKRNRKDSEVD	47.6655	3.9	2
DMEDMCVDAMDDMVDMKDDM	36.1302	1.2	1.9
EKDKKVKVKKDKKDMRDEKD	42.9125	3.9	2
EEMEDEWVEVKVDWKDEYME	43.9405	3.9	2
KMVKEAAEEQMSDMDEKVDR	33.3266	1.4	1.9
DDRMDKVMKDMKDDMDDMMD	45.231	1.3	2
DMMEDMMDDMVDVKDDKMDK	46.3153	1.3	1.9

KDWKDDKVEKMDKEMKDMKD	54.9607	3.9	2
VDEMDDMMDVKVDAMDDKMD	54.2886	3.9	1.9
DMMSDMKDDMEDMMDEMDKK	54.806	3.9	2
MRDKKDKEDREMKDKRDKMD	53.9301	3.1	2
VKDDMEDMAMDMKDDMMDEM	39.5695	1.3	1.9
REDMENEMRDMKDDMEMEYK	40.3428	1.4	2
DRMDVMDDMMMDKDERMEKE	40.8571	1.3	2
MKADMYDDAMVDKDDRMERE	35.9201	1.3	1.9
KEERDMMDDKMDVMDEMYEK	44.1841	1.3	2
KDEKDKKERMKRDEKDEKLK	48.6624	3.9	1.9
KKQEKIKKEKKDMKEEEMKD	34.0361	1.5	1.9
KDQKMDKVDRKDKMMEDKEE	49.1149	3.9	2
FIKDEIDKADEEMWERYDKE	42.7002	1.4	1.9
CKEDEKSRKRNRVENREEKE	38.4889	3.9	2
IKDEMDKQERDKKERREKKD	48.1378	3.9	2.1
RDDRKNKERKDRKERKDREE	47.3495	3.1	2.2
KDKEDMKMEQMYEDWDDLMD	54.4551	3.9	1.9
EDIKDMIDDIIMDIKDEKDK	44.2311	1.4	1.9
DEYMEWLKDKEDKYDKWERD	48.9954	3.9	2
KEIIDEIMDEIEDKMEMMEE	43.0757	1.2	1.9
KEKKDREEKELEDMIEKIKE	45.8599	2.3	1.9
DEDSKARKRRRREDKDREDR	35.2293	1.9	2.1
DYRDEKMDMREMMDEEEDRM	52.6067	3.9	2
EMMEEDKMDMIEDKIDKKME	41.9646	1.3	1.9
EMIRDMEEEIMEDIEDKMER	45.9471	1.4	1.9
EERRYEEWYMDEFEDEEYKK	46.739	3.9	1.9

Table S1: List of sequences with selective binding towards POPE:POPG membrane.

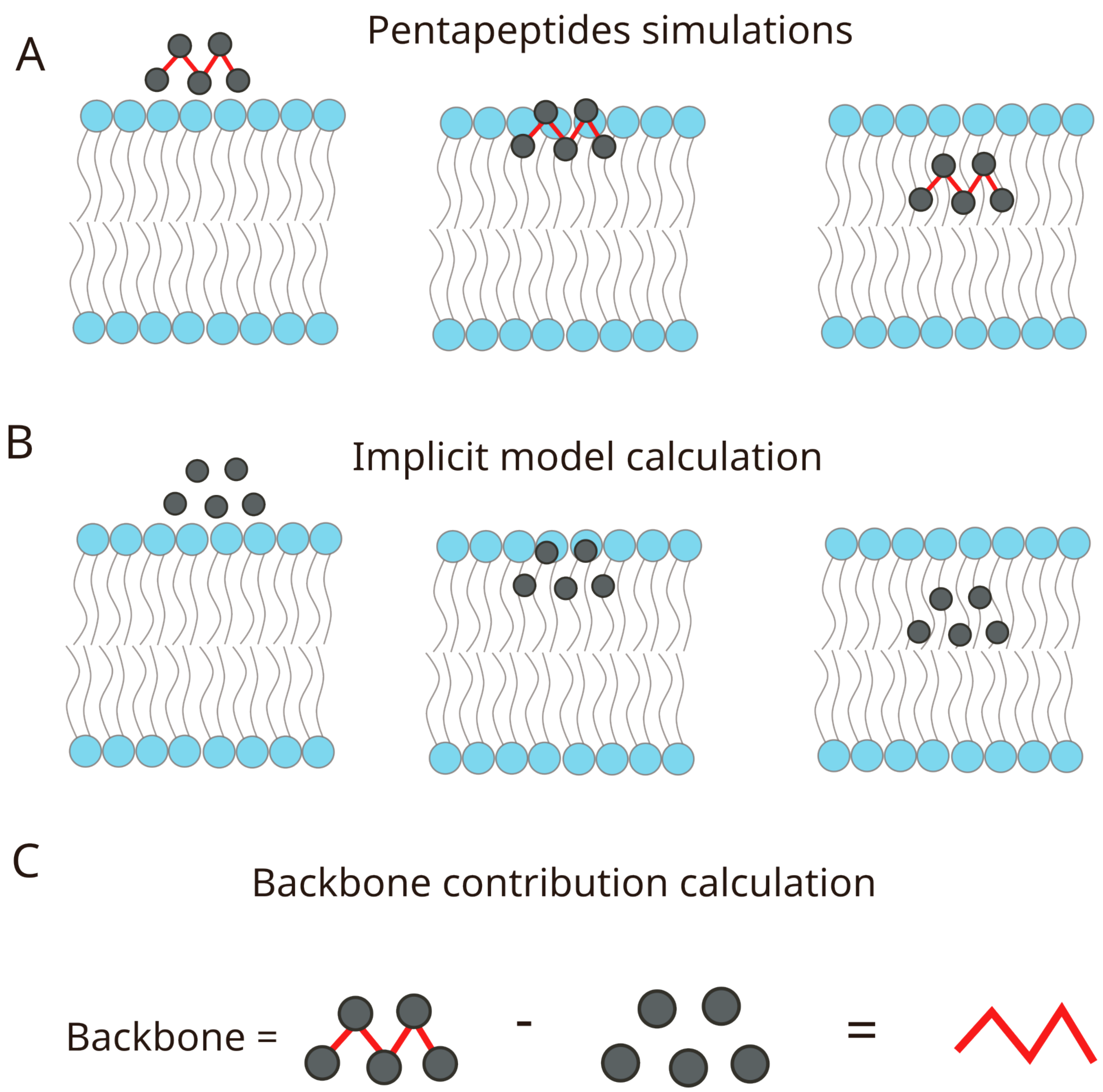

Figure S2: A) Schematic of the pentapeptides simulations. The gray balls represent the side chains and the red line the backbone of the peptide. The peptide was kept parallel to the membrane plane throughout the pulling inside of the membrane. B) Schematic of the calculation of the side chain contributions from the implicit model. To calculate the backbone contribution we also kept the peptide in the implicit model parallel to the membrane. C) Calculation of the backcbone contribution was made by subtracting the PMF obtained from the pentapeptide simulations and the PMF from the side chains.

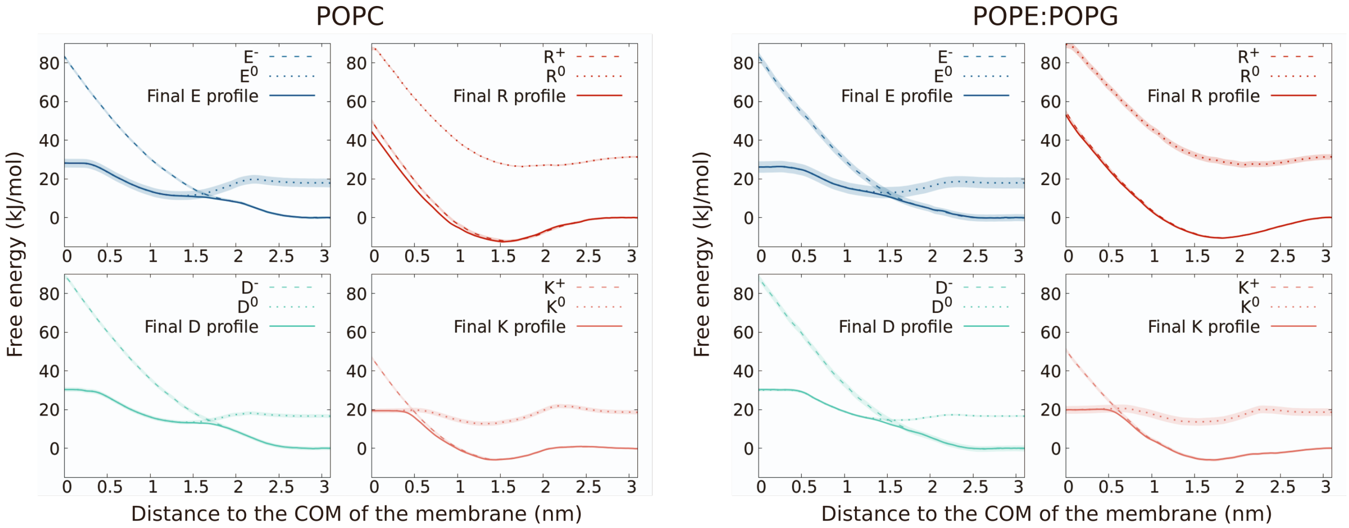

Figure S3: PMFs of charged side chains. The final PMF is calculated from the Boltzmann average between the neutral and charged states.

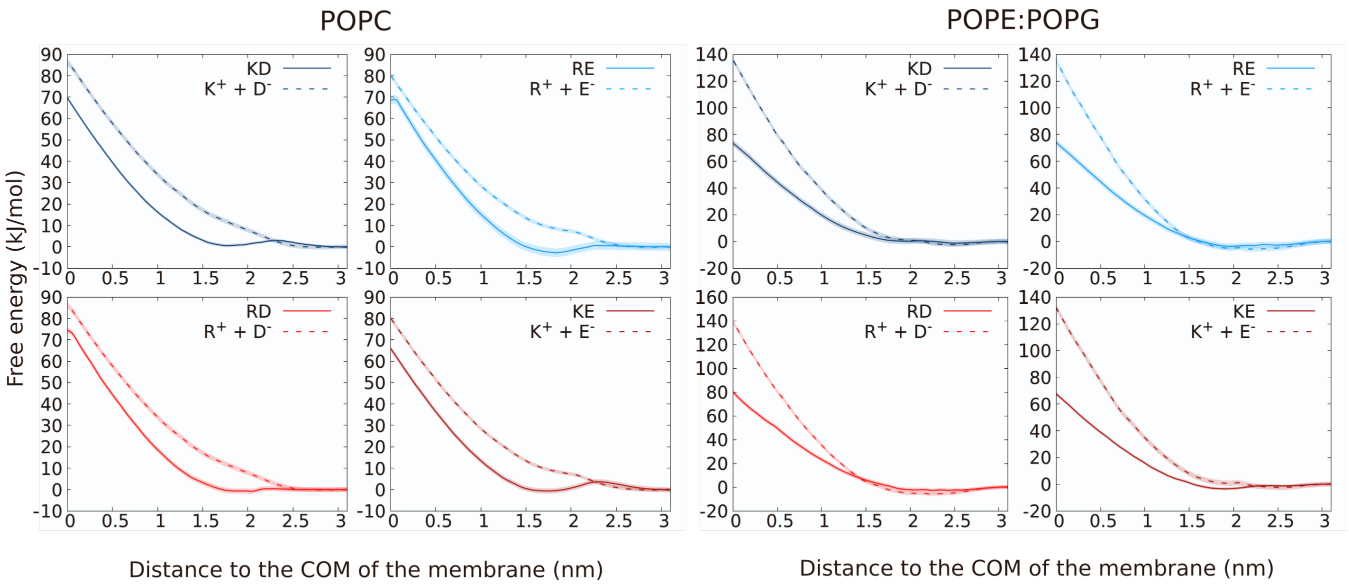

Figure S4: Comparison of PMFs of charged aminoacids forming salt-bridges. KD, RD, KE, RE are the PMFs obtained from simulating the side chains at a close distance, enough for them to interact. $K^+ + D^-$, $R^+ + D^-$, $K^+ + E^-$, $R^+ + E^-$ are the sum of the contributions of the single side chains simulated individually.

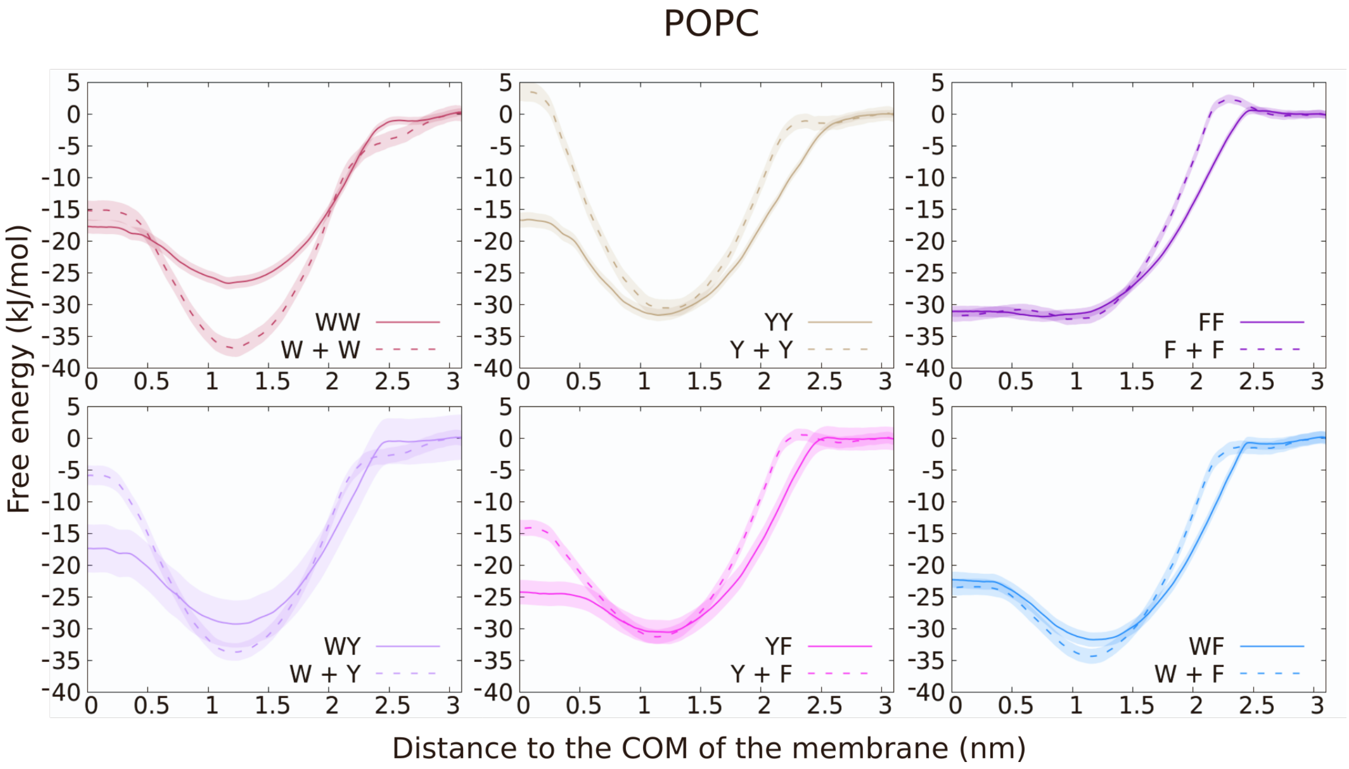

Figure S5: Comparison of PMFs in POPC of aromatic aminoacids interacting. WW, YY, FF, WY, YF, WF are the PMFs obtained from simulating the side chains at a close distance, enough for them to interact. $W+W$, $Y+Y$, $F+F$, $W+Y$, $Y+F$, $W+F$, are the sum of the contributions of the single side chains simulated individually.

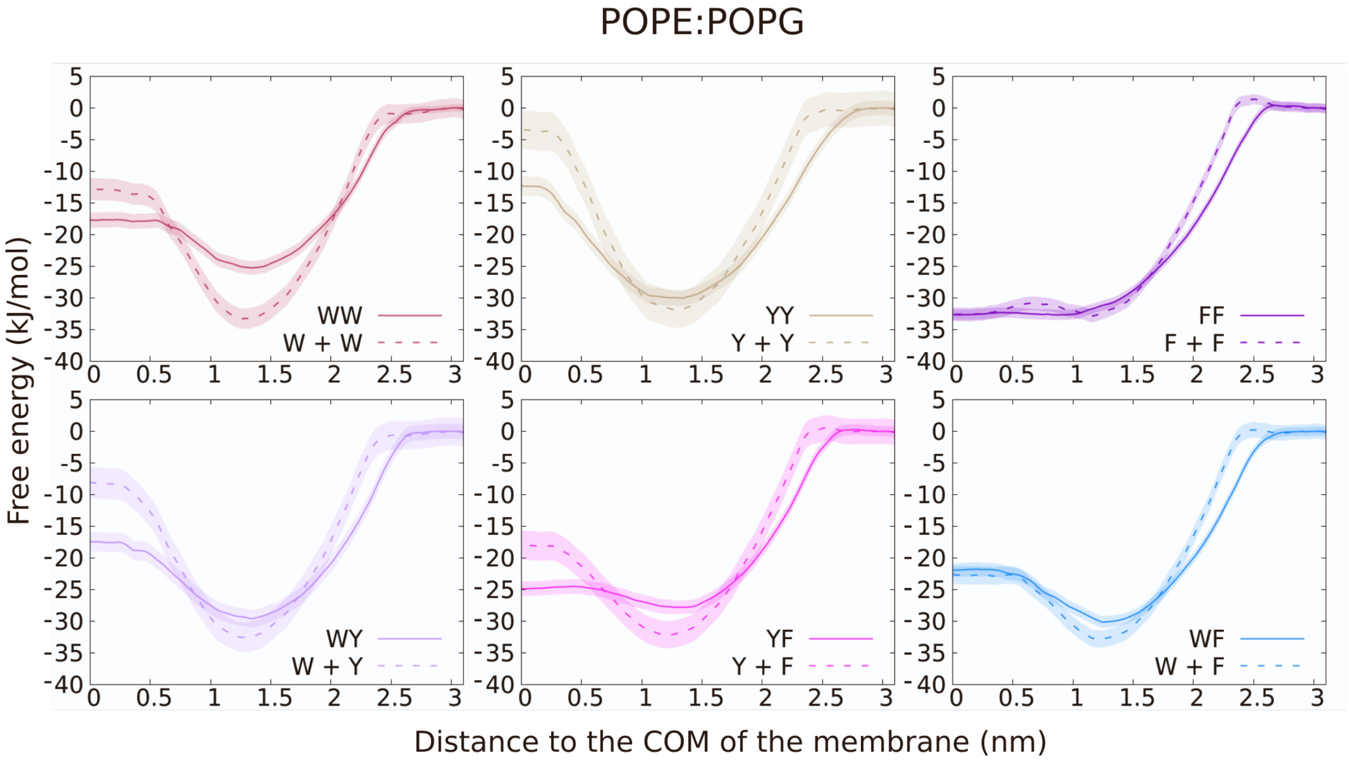

Figure S6: Comparison of PMFs in POPE:POPG of aromatic aminoacids interacting. WW, YY, FF, WY, YF, WF are the PMFs obtained from simulating the side chains at a close distance, enough for them to interact. $W+W$, $Y+Y$, $F+F$, $W+Y$, $Y+F$, $W+F$, are the sum of the contributions of the single side chains simulated individually.

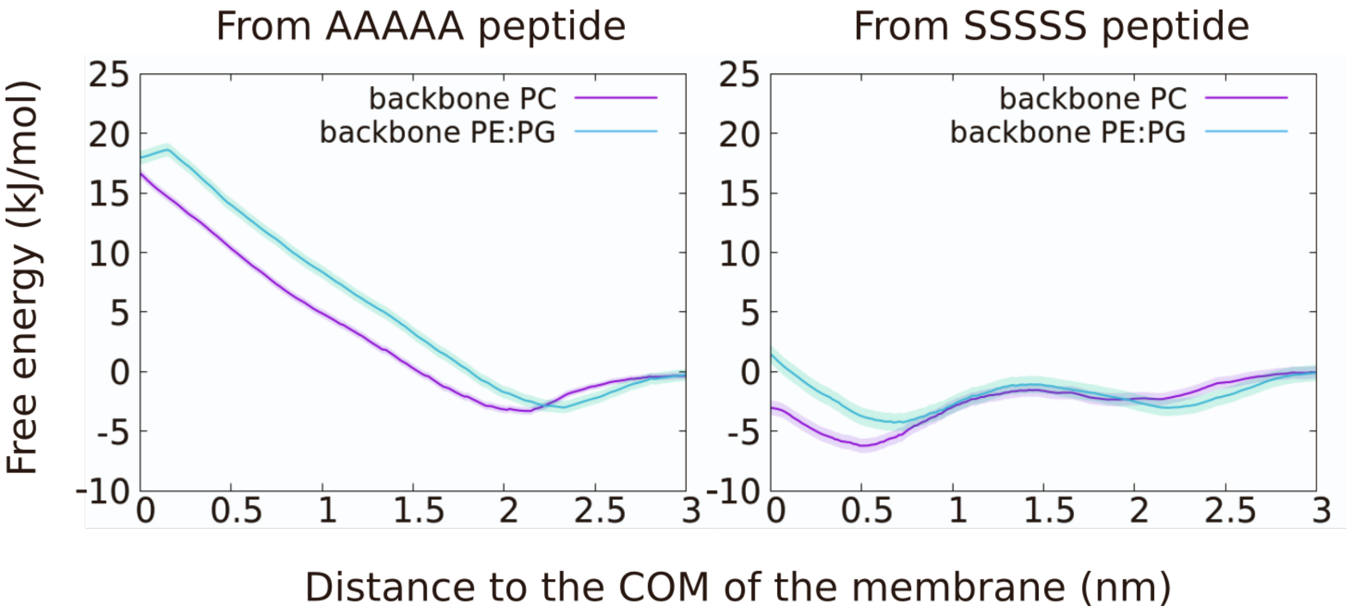

Figure S7: Backbone contributions calculated from pentapeptides of alanine and serine. The results are obtained from subtracting the implicit model PMF to the PMF of the all-atom simulation and dividing by 5 to obtain the backbone contribution of a single residue.

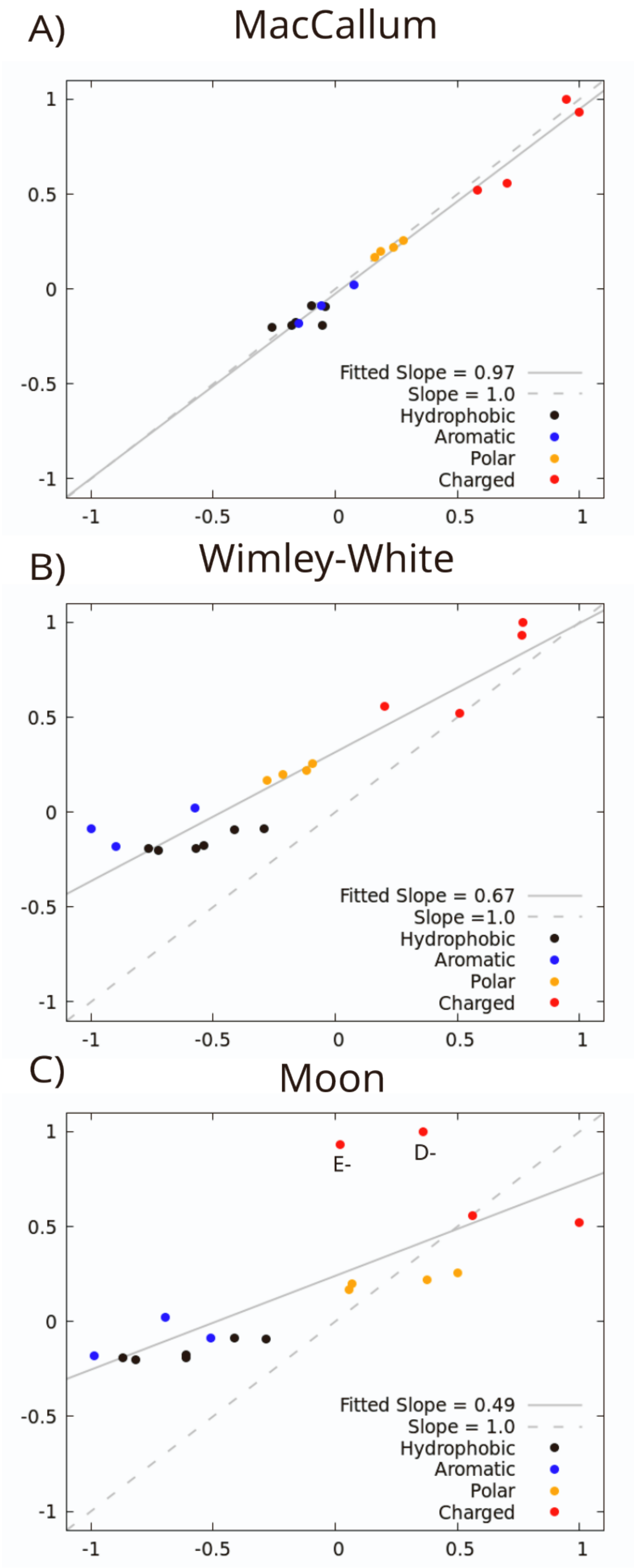

Figure S8: A) Comparison between our results in POPC membrane and MacCallum hydrophobicity scale also obtained from molecular dynamics. B) Comparison between our results and Wimley-White hydrophobicity scale obtained experimentally from residues partition between water and octanol. C) Comparison between our results and Moon hydrophobicity scale. Our results correlate well with the three scales, with $R^2 = 0.9807$ for the MacCallum scale, $R^2 = 0.8744$ for the Wimley-White scale, and $R^2 = 878$ for the Moon scale after removing the outliers (labeled in the plot as E- and D-).

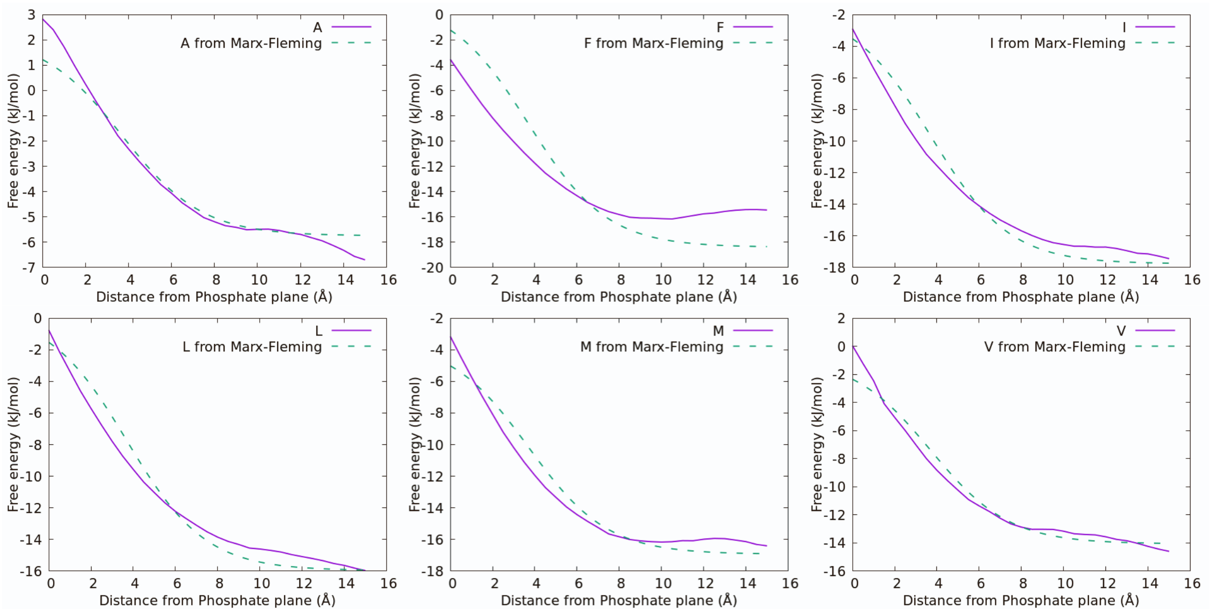

Figure S9: Comparison between free energy profiles from side chain simulations at POPC membrane and Marx-Fleming model of free energy in the interface region of the bilayer [D.C. Marx and Karen G. Fleming. Journal of the American Chemical Society, 143(2):764–772, January 2021.].

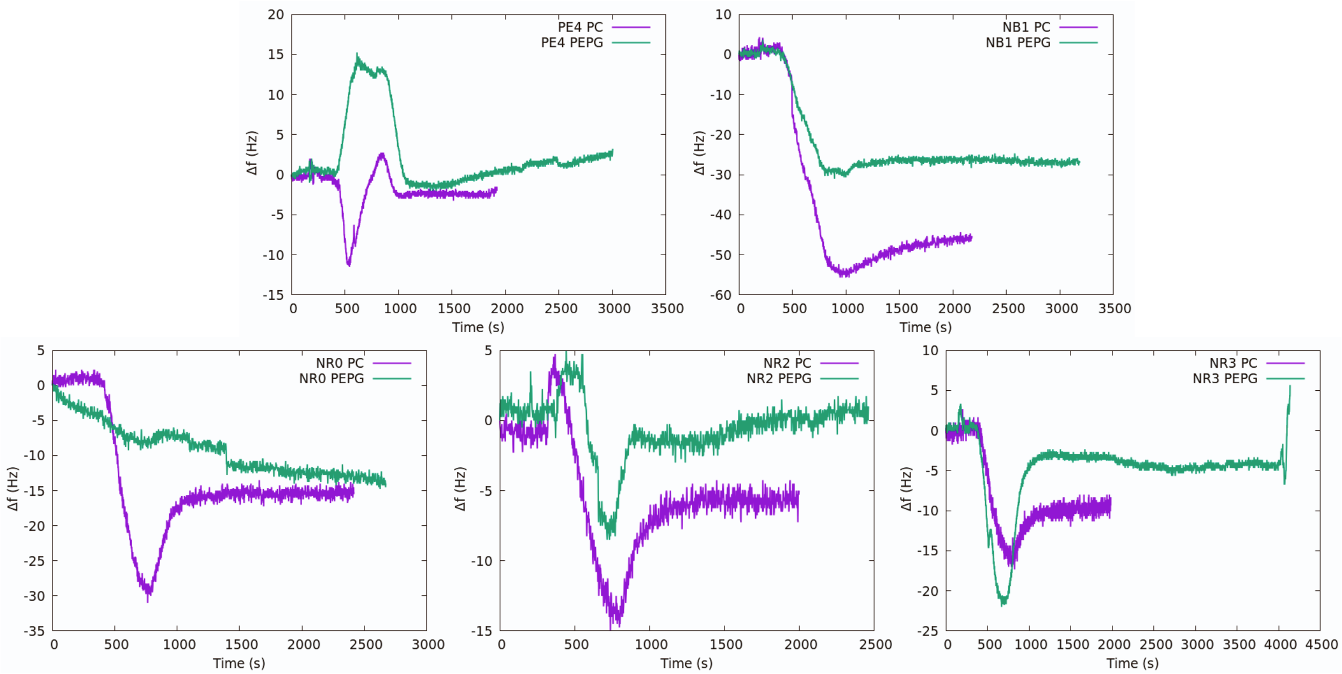

Figure S10: Peptides with predicted selectivity towards PEPG that do not show preferential binding to PEPG vesicles in QCM experiments. Peptide was added at 200 s. The decrease in frequency measures the amount of peptide bound to the bilayers. The data shown is the average of four (NR0, PE4) or three replicas (NB1, NR2, NR3) and only the fifth overtone has been considered for simplicity. Both x and y axis are shifted so that 0 represents the stable bilayer without peptide.

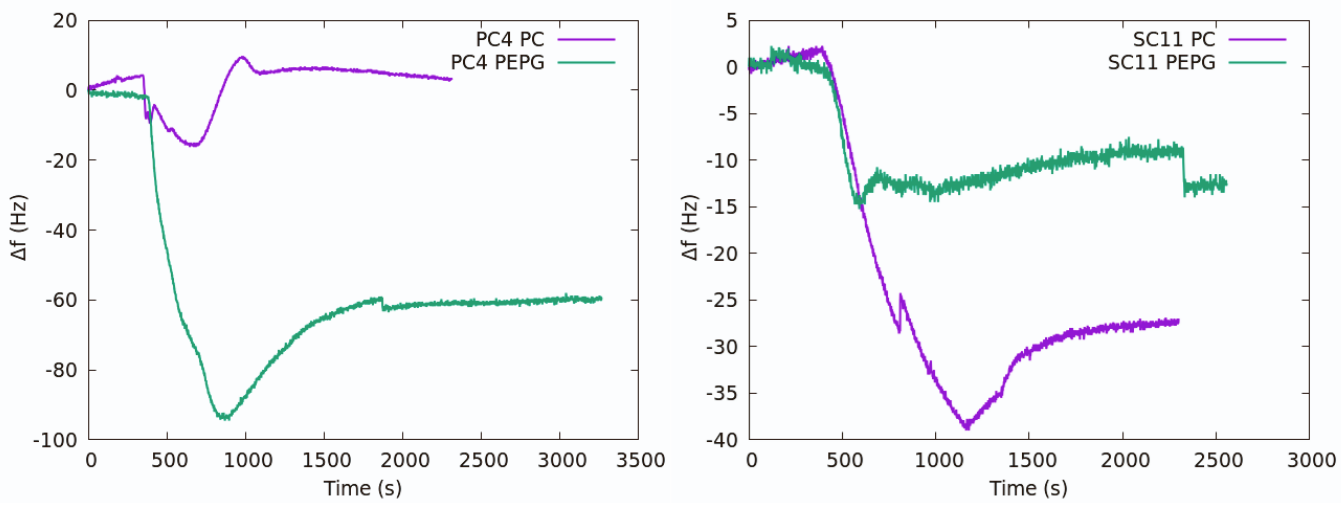

Figure S11: QCM results of PC4 with predicted selectivity to POPC membranes, and SC11 with small to none selectivity predicted. In these cases, the implicit model prediction does not match the QCM results. Peptide was added at 200 s. The decrease in frequency measures the amount of peptide bound to the bilayers. The data shown is the average of four replicas and only the fifth overtone has been considered for simplicity. Both x and y axis are shifted so that 0 represents the stable bilayer without peptide.

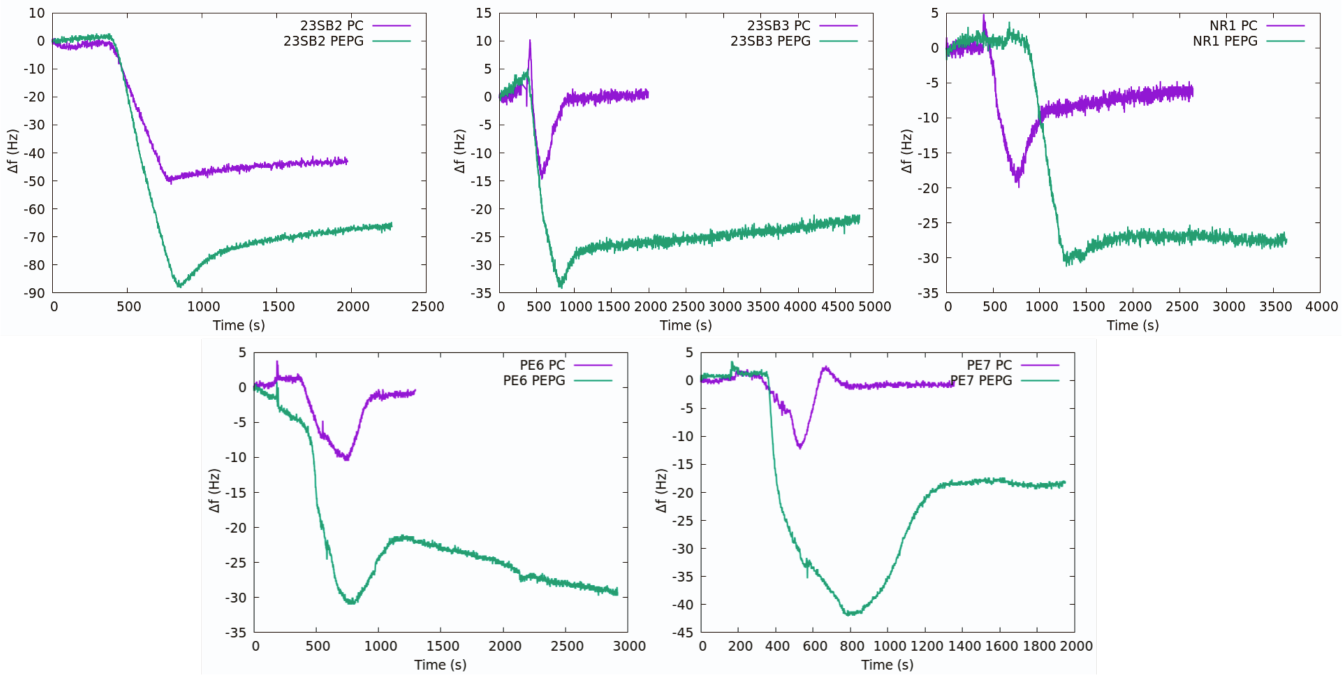

Figure S12: Peptides with predicted selectivity towards PEPG that also show preferential binding to PEPG vesicles in QCM experiments. Peptide was added at 200 s. The decrease in frequency measures the amount of peptide bound to the bilayers. The data shown is the average of four replicas and only the fifth overtone has been considered for simplicity. Both x and y axis are shifted so that 0 represents the stable bilayer without peptide. The plots display an average over the three (23SB2 and PE7) or four replicas (23SB3, NR1, and PE6).

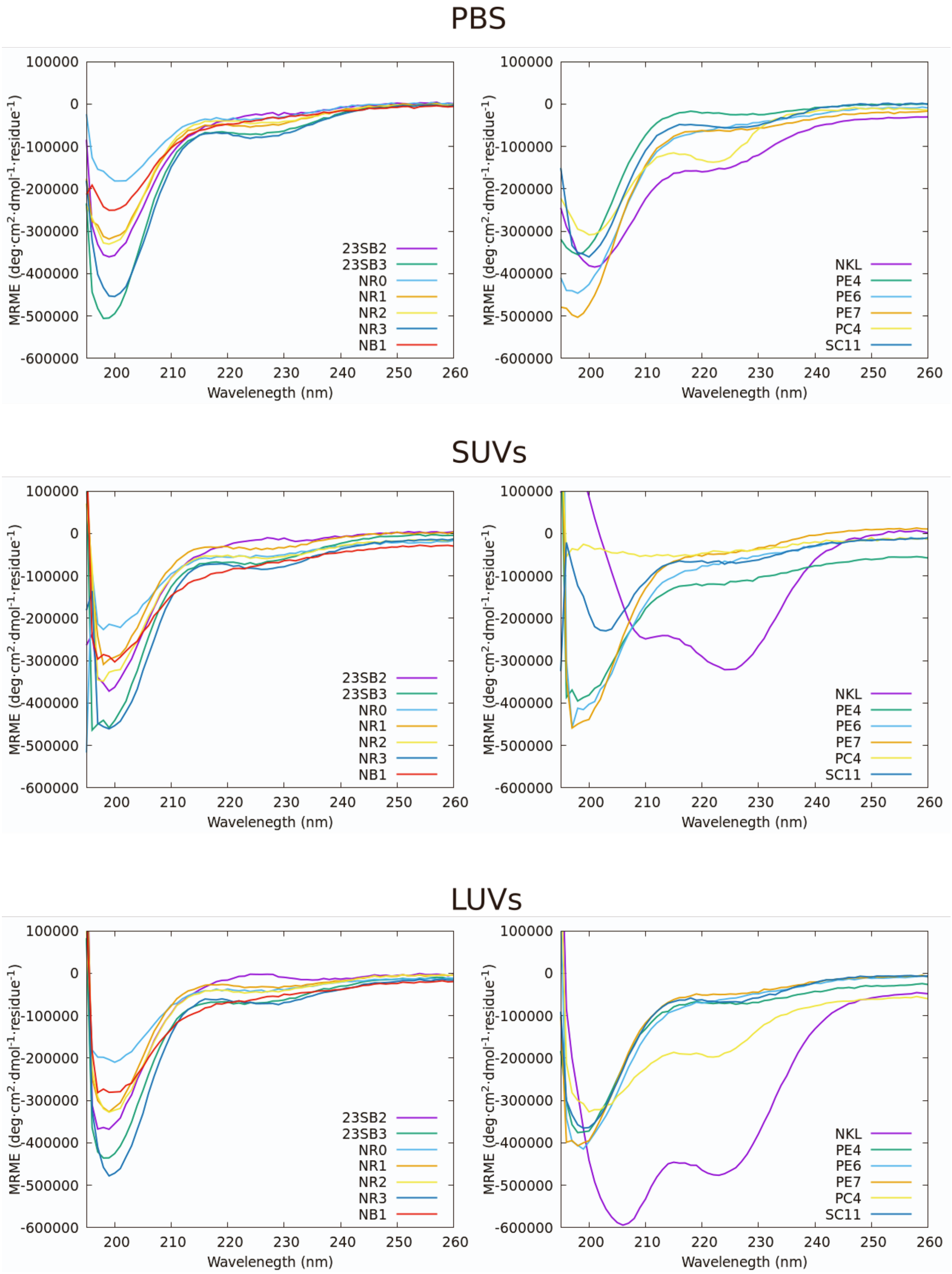

Figure S13: CD spectra of the peptides tested in solution and in the presence of LUVs. Peptides with alpha helical secondary structure show characteristic decreases in mean residue molar ellipticity (MRME) at 210 nm and 225 nm. In contrast, peptides in random coil conformation show a decrease of signal at 200 nm. The only peptide with clear alpha helical conformation is NKL when measured in the presence of LUVs or SUVs.

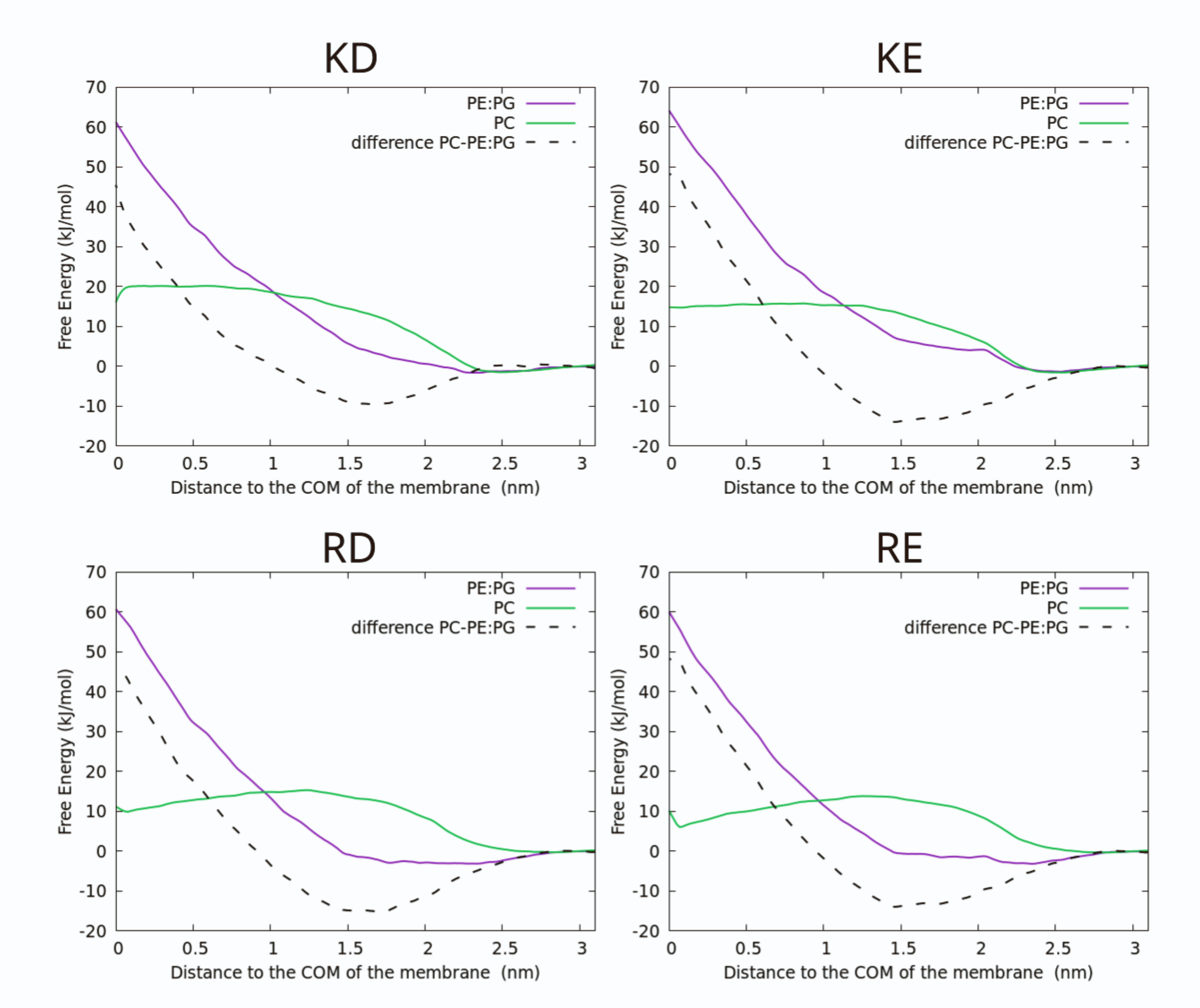

Figure S14: The figure shows the effect of the side chain correlations of charged residues forming salt-bridges in each of the membranes. The dotted black line shows the big differences that these correlations cause between membranes.

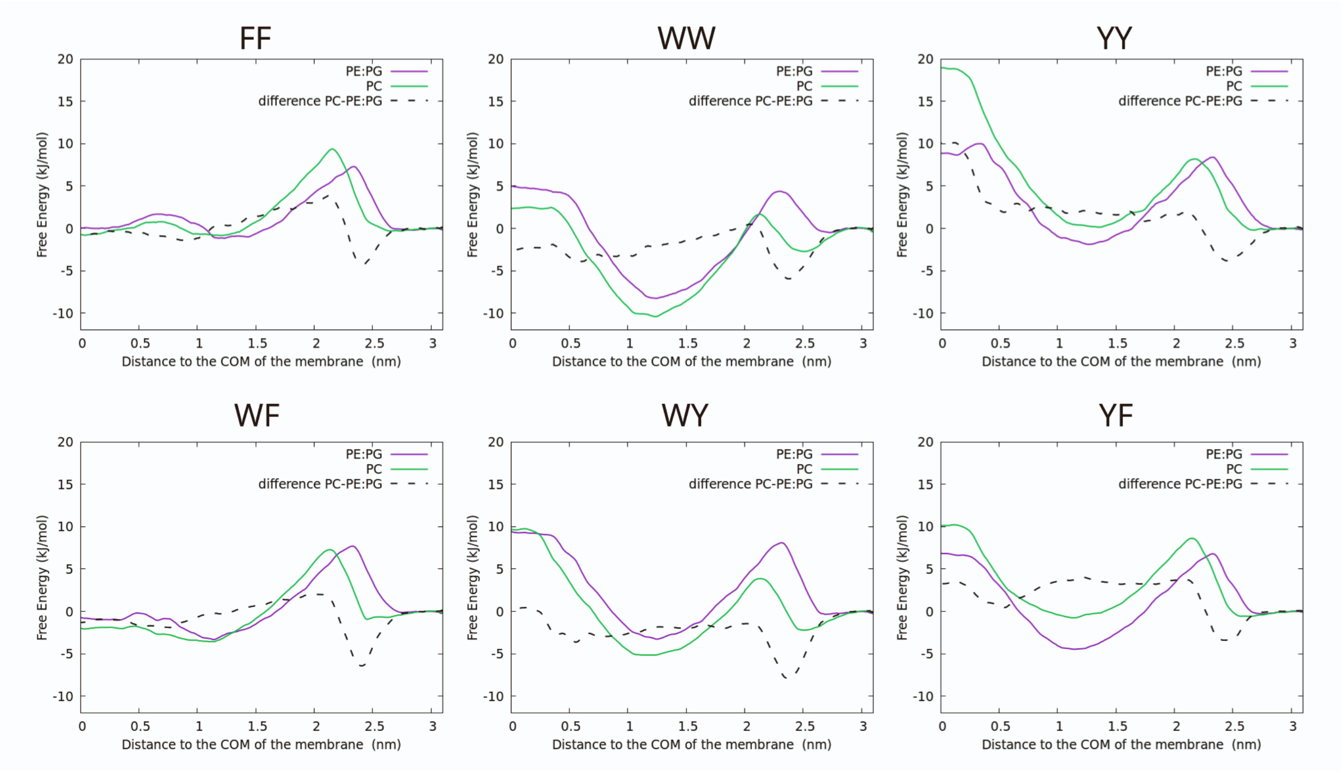

Figure S15: The figure shows the effect of the side chain correlations of aromatic residues in each of the membranes. The dotted black line shows the smaller differences that these correlations cause between membranes.

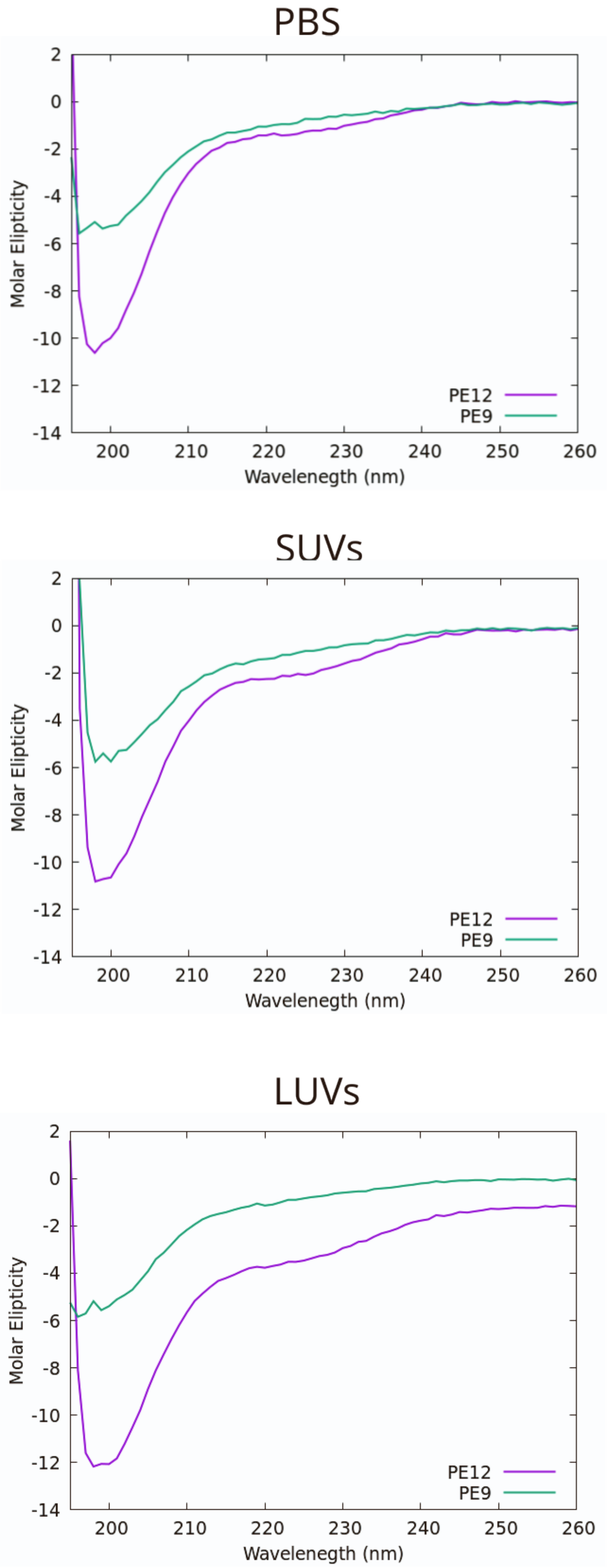

Figure S16: CD spectra of peptides PE9 and PE12 tested in PBS and in the presence of LUVs/-SUVs. Peptides with alpha helical secondary structure would show characteristic decreases in mean residue molar ellipticity (MRME) at 210 nm and 225 nm. In contrast, peptides in random coil conformation show a decrease of signal at 200 nm. Both PE9 and PE12 are therefore unstructured in PBS solutions and in the presence of LUVs or SUVs.

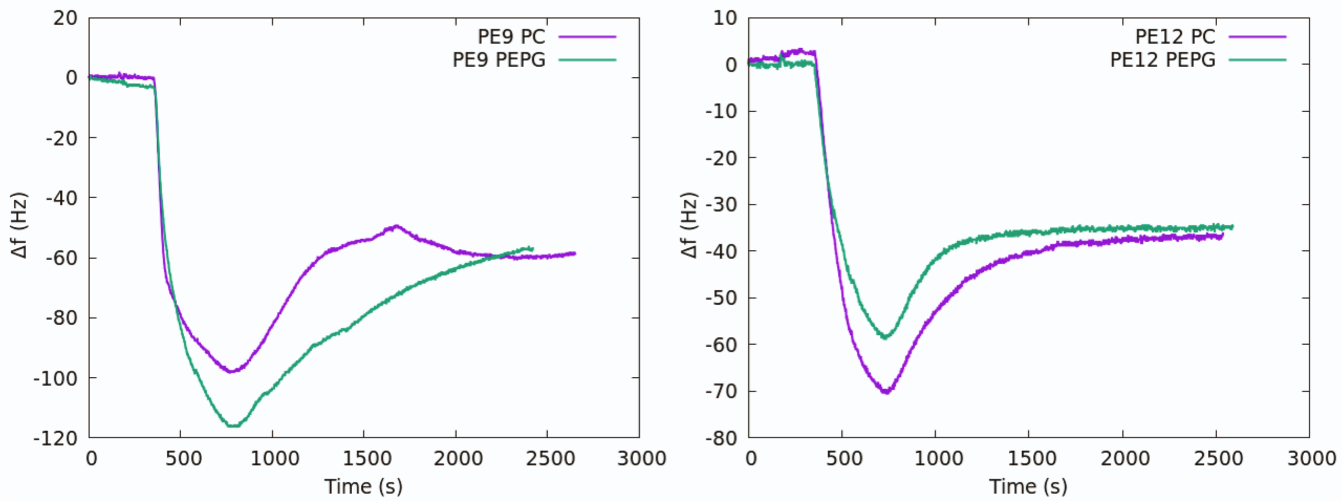

Figure S17: QCM experiments of peptides PE9 and PE12. Peptides were added at 200 s. The decrease in frequency relates to the amount of peptide bound to the bilayers. The data shown is the average of two replicas. Only the fifth overtone has been considered for simplicity. Both x and y axis are shifted so that 0 represents the stable bilayer without peptide.
